# Selective Localization of Shanks to VGLUT1-Positive Excitatory Synapses in the Mouse Hippocampus

**DOI:** 10.3389/fncel.2016.00106

**Published:** 2016-04-26

**Authors:** Christopher Heise, Jan C. Schroeder, Michael Schoen, Sonja Halbedl, Dominik Reim, Sarah Woelfle, Michael R. Kreutz, Michael J. Schmeisser, Tobias M. Boeckers

**Affiliations:** ^1^Institute for Anatomy and Cell Biology, Ulm UniversityUlm, Germany; ^2^RG Neuroplasticity, Leibniz Institute for NeurobiologyMagdeburg, Germany; ^3^Department of Neurology, Ulm UniversityUlm, Germany

**Keywords:** Shank1, Shank2, Shank3, synapse, VGLUT1, VGLUT2, hippocampus, mossy fibers

## Abstract

Members of the Shank family of multidomain proteins (Shank1, Shank2, and Shank3) are core components of the postsynaptic density (PSD) of excitatory synapses. At synaptic sites Shanks serve as scaffolding molecules that cluster neurotransmitter receptors as well as cell adhesion molecules attaching them to the actin cytoskeleton. In this study we investigated the synapse specific localization of Shank1-3 and focused on well-defined synaptic contacts within the hippocampal formation. We found that all three family members are present only at VGLUT1-positive synapses, which is particularly visible at mossy fiber contacts. No costaining was found at VGLUT2-positive contacts indicating that the molecular organization of VGLUT2-associated PSDs diverges from classical VGLUT1-positive excitatory contacts in the hippocampus. In light of SHANK mutations in neuropsychiatric disorders, this study indicates which glutamatergic networks within the hippocampus will be primarily affected by shankopathies.

## Introduction

The *SHANK* gene family comprises *SHANK1, SHANK2*, and *SHANK3* and encodes for PSD-associated scaffolding proteins at the excitatory synapse that interconnect neurotransmitter receptors and cell adhesion molecules by direct and indirect interactions with numerous other PSD-associated proteins (Sheng and Kim, 2000; Boeckers et al., 2002; Grabrucker et al., 2011b; Sala et al., 2015). Several studies have shown that Shanks are important molecules for proper excitatory synapse and circuit function (Peca et al., 2011; Schmeisser et al., 2012; Jiang and Ehlers, 2013). Interestingly, mutations in *SHANKS*—especially in *SHANK3* and *SHANK2*—have been associated with neuropsychiatric disorders, in particular autism spectrum disorder (ASD) (Leblond et al., 2014). In ASD, growing evidence points toward alterations in neuronal circuits (Zikopoulos and Barbas, 2013; Gogolla et al., 2014; Rothwell et al., 2014) and ASD also appears to be associated with a diminished interaction of the hippocampal and orbitofrontal systems (Ameis et al., 2011). The reduced functional coupling represents one of the possible underpinnings of abnormal socio-emotional behavior observed in ASD patients since the hippocampus modulates cortex-dependent behaviors by engaging in memory retrieval relevant to the situation the organism is facing (Ramus et al., 2007; Lehn et al., 2009; Pehrs et al., 2015). The hippocampus receives input mainly from the entorhinal cortex (EC) within the temporal lobe and processes the information by relaying the signals within the trisynaptic circuit. The EC projects via the perforant path to the stratum moleculare of the dentate gyrus (DG) where dendritic spines of granule cells receive the excitatory input. These cells then transduce the excitatory input along mossy fibers (MFs) to the stratum lucidum/stratum radiatum in CA3, where giant MF contacts exist on large dendritic spines (also known as thorny excrescences) of pyramidal cells. Of note, the axons of the granule cells also form several large excitatory synaptic contacts with mossy cells and other interneurons in the intragranular mossy fibers (IMF), i.e., in regions of the MF proximal to the DG. CA3 pyramidal cells, in turn, project to the stratum radiatum in CA1 via Schaffer collaterals where they form contacts with CA1 pyramidal cells which then project back to the EC via the subiculum.

The vesicular glutamate transporters (VGLUTs) VGLUT1 and VGLUT2 are critical for excitatory signal transmission (Fremeau et al., 2004; Wojcik et al., 2004) and in some knock-out lines genetic deletion of VGLUT1 and VGLUT2 has resulted in premature death (Moechars et al., 2006; Balschun et al., 2010). VGLUT3 is less clearly associated with excitatory neurotransmission and rather functions at presynaptic terminals of neurons mediating modulatory/monoaminergic signals (Gras et al., 2002; Schafer et al., 2002; Amilhon et al., 2010; Soiza-Reilly and Commons, 2011). Generally speaking, VGLUT1, and VGLUT2 display a complementary expression profile in the CNS of rodents and humans (Fremeau et al., 2001, 2004; Varoqui et al., 2002; Commons et al., 2005; Vigneault et al., 2015) and segregate input from different brain regions. In the hippocampus, VGLUT1-positive presynaptic terminals originate mainly from neurons within the hippocampus itself and from the cortex (Balschun et al., 2010; Zander et al., 2010), whereas the origin of VGLUT2-positive presynaptic terminals is the supramammillary nucleus (SUM) or mossy cells from other regions of the hippocampus (Halasy et al., 2004; Boulland et al., 2009).

Here we report that in the mouse hippocampus Shank1-3 exclusively reside at VGLUT1-positive synapses—which is particularly visible along the MFs—and not at VGLUT2-positive synapses. Our data suggests that excitatory scaffolds can vary significantly in their genetic makeup even within a single brain region, and identifies the postsynapses of VGLUT2-positive synapses as Shank-negative in the hippocampus. Considering the strong link between genetic alterations of human *SHANK* and neuropsychiatric disorders, our study suggests that VGLUT1-dependent neuronal networks within the hippocampus (e.g., the trisynaptic circuit) may be primarily affected by shankopathies.

## Materials and methods

### Animal ethics statement

Shank2^−/−^ and Shank3αβ^−/−^ mice were previously described (Schmeisser et al., 2012). All mice were kept in specific pathogen-free animal facilities and all animal experiments in this study were performed based on the guidelines for the welfare of experimental animals issued by the Federal Government of Germany and by the local ethics committee (Ulm University), ID Number: 0.103.

### Vector constructs

Full length rat GFP-Shank1A was a kind gift of Dr. Carlo Sala and has been previously described (Romorini et al., 2004). Full length rat GFP-tagged Shank2 (Boeckers et al., 2005), full length human mcherry-tagged GFP-Shank2 (Peykov et al., 2015), full length rat GFP-Shank3a (Grabrucker et al., 2011a), and human GFP-Shank3a (Cochoy et al., 2015) have been previously described, as well.

### Primary antibodies

Primary antibodies used for immunocytochemistry were all diluted 1:200, for western blotting a dilution of 1:500 was used (except for actin which was diluted 1:100000). The Shank2 (“ppI-SAM pabSA5192”) and Shank3 antibodies (“PRC pab,” simply referred to as “Shank3” in the study and “C-term/ProSAP2/Shank3”) have been characterized previously (Bockers et al., 1999; Bockmann et al., 2002; Schmeisser et al., 2012). The following primary antibodies were purchased from commercial suppliers: Actin (Sigma-Aldrich Cat# A2228 RRID:AB_476697), Bassoon (Enzo Life Sciences Cat# ADI-VAM-PS003-F RRID:AB_11181058), CTIP2 (Abcam Cat# ab18465 RRID:AB_2064130) GAD65 (Abcam Cat# ab85866 RRID:AB_1860505), Homer 1b/c (Synaptic Systems GmbH Cat# 160 023, no RRID yet), GluN1 (Sigma-Aldrich Cat# G8913 RRID:AB_259978), Shank1 (Novus Cat# NB300-167 RRID:AB_2187584), SPO (Synaptic Systems GmbH Cat# 102 002 RRID:AB_887841), Syn1/2 (Synaptic Systems GmbH Cat# 160 003, no RRID yet), VGLUT1 (Synaptic Systems GmbH Cat# 135 304 RRID:AB_887878), VGLUT2 (Synaptic Systems GmbH Cat# 135 404 RRID:AB_887884), and VGLUT 3 (Synaptic Systems Cat# 135204, no RRID yet).

### Secondary antibodies

Secondary antibodies used in immunocytochemistry were all coupled to Alexa Fluor^®;^ 488, 568, or 647 (all from Life Technologies, dilution 1:500). Secondary antibodies used for western blotting were HRP-conjugated secondary antibodies (Dako, Glostrup, Denmark, dilution 1: 1000).

### Cell culture

HEK293T were kept in DMEM at 37°C in 5% CO_2_ as previously described (Cochoy et al., 2015).

### Transfection

HEK293T were transfected as previously described (Cochoy et al., 2015).

### Section preparation and immunohistochemistry

Animals were anesthesized (ketamine 100 mg/kg and Xylazin 16 mg/kg, solubilized in an NaCl solution) and perfused with 25 ml cooled PBS and 50 ml paraformaldehyde 4%. Then, brains were left overnight in 4% paraformaldehyde, followed by an incubation in 30% sucrose. Finally, brains were frozen in OCT compound and put at −80°C until the day before cryostat sectioning. One day before sectioning, brains were put at −20°C to adapt to the cutting temperature (−22°C) of the sectioning procedure. 40 μm coronal sections were then made using a cryostat (Leica CM3050 S) and appropriate microtome blades (Feather, A35 Type). Sections were then transferred to PBS without calcium and magensium (PBS^−/−^) for free floating antibody labeling: Incubation with blocking solution (3% BSA + 0.3% Triton-X-100, diluted in PBS^−/−^) took place for 2 h at RT on horizontal shaker. Then, sections were transferred to wells containing the primary antibody (dilution 1:200 in blocking solution) and incubation took place for 48 h at 4°C on horizontal shaker. 3 washes in PBS^−/−^ (30 min at RT on horizontal shaker) and the incubation with fluorophore conjugated secondary antibodies (all coupled to Alexa Fluor^®;^ 488, 568 or 647 (all from Life Technologies), dilution 1:500) for 2 h at RT on horizontal shaker followed. Then, 3 washes in PBS^−/−^ (30 min at RT on horizontal shaker) followed and mounting took place using mowiol containing diluted 4,6-diamidino-2-phenylindole DAPI (dilution 1:50000). Images were either captured using an upright fluorescence microscope (view 2.7.1 for details) and Axiovision software (Zeiss) for the 5X overview images or using a confocal microscope (view 2.7.1 for details) using Zeiss proprietary software LSM5 the for the close-up images. Image processing took place with ImageJ (US National Institutes of Health). View “2.7.1 Image analysis and image selection for immunocytochemical analysis” for further details.

#### Image analysis and image selection for immunocytochemical analysis

Qualitative immunocytochemical analysis was carried out on 5 male adult mice (age: 2 months). For quantitative immunocytochemical analysis 5–8 images from these mice were selected. At least 3 coronal sections were stained for each antibody/antibody combination per animal to assure representativeness of stainings. For confocal microscopy, an LSM 710 microscope from Zeiss was used (objective Plan-Apochromat 63x/1.40 Oil DIC M27; numeric aperture 1.40, a pinhole setting which represents one airy disc was used; used lasers had an excitation wavelength of 405 nm, 488, 561 and 633 nm with emission filters of 410–488 nm, 493–551 nm, 570–628 nm, and 638–747 nm, respectively). For confocal microscopy, the acquisition depth in the z-axis was 3 μm at acquisition intervals of 0.5 μm, i.e., 7 planes per coronal section. Stainings were assessed plane by plane and representative 1 μm maximum projections were created using ImageJ. For wide field microscopy pictures were taken with an upright Axioscope microscope equipped with a Zeiss CCD camera (objective a Plan-NEOFLUAR 5x/0.15 with NA 0.15; light source: ebq 100 isolated; filters: BP 515-565, BP 575-640, BP 690/50).

For quantitative immunocytochemical analysis of the Shank1-3/VGLUT1-2 colocalization, two 63x images at acquisition intervals of 0.5 μm were analyzed by the image software Imaris. Analysis either pertained to entire 63x image (192.8 μm × 192.8 μm) or to a subfield (48.2 μm × 48.2 μm) within the 63x image (view also Supplementary Figures 2, 2 and 2, 3). Puncta were identified by Imaris using the “quality” parameter and only puncta stringently conforming with the quality parameter (bottom 10%) entered colocalization analysis. The center of mass of the identified puncta was allowed a maximal distance of 0.7 μm. Statistical analysis was carried out using an ANOVA with a *post-hoc* tukey test.

### Biochemistry

HEK293T were transfected with full length rat GFP-Shank1A, rat GFP-tagged Shank2, human mcherry-tagged GFP-Shank2, rat GFP-Shank3a, human GFP-Shank3a, or appropriate empty vectors. Lysis of transfected HEK293T was carried out in Triton X-100 Lysis Buffer (150 mM NaCl, 50 mM Tris HCl, 1% Triton X-100, pH 7.4, protease inhibitor mix from Roche).

Crude synaptosomal fractions from mouse hippocampus were biochemically isolated as described previously (Schmeisser et al., 2012). In brief, hippocampal tissue from male wildtype mice was homogenized in HEPES-buffered sucrose (320 mM sucrose, 5 mM HEPES, pH 7.4) containing protease inhibitors (Roche). Homogenates were centrifuged at 1000 g for 10 min at 4°C to pellet cell debris and nuclei. The pellet was discarded while the supernatant was then centrifuged at 12,000 g for 20 min at 4°C to yield the pelleted crude synaptosomal fraction which was then solubilized in HEPES-buffered sucrose.

Both the HEK cell lysate and the crude synaptosomal fractions from the hippocampus were then analyzed by Bradford assay to assess protein concentration. Samples were diluted by 4x loading dye (200 mM Tris-HCL, pH 6.8, 200 mM DTT, 4% SDS, 4 mM EDTA, 40% glycerol, 0.02% bromophenolblue) to a final concentration of 1 μg/μl. For western blotting, equivalent amounts of protein were loaded per lane (15 μg for HEK cell lysates and 25 μg for crude synaptosomal fractions from mouse hippocampus). Western blot analysis was carried out according to standard protocols. HRP-conjugated secondary antibodies (Dako, Glostrup, Denmark) were used in combination with the SuperSignal detection system (Thermo Scientific) to visualize protein bands.

## Results

### Shank1-3 distribution in the mouse hippocampus using specific antibodies

In this study we used target specific Shank antibodies which have been extensively characterized in a recent work (Halbedl et al., 2016) and were used in previous studies. For Shank1 we used NB300-167 from Novus (Schmeisser et al., 2012), for Shank2 we used the “ppI-SAM pab SA5192” antibody (Bockers et al., 1999; Schmeisser et al., 2012), for Shank3 we primarily used the “PRC pab” antibody (Schmeisser et al., 2012), and in one case (Supplementary Figure S4) the “Shank3 (C-term)” antibody (Bockmann et al., 2002) was used (Supplementary Figure S1A). Initially, we expanded the specificity analysis of the Shank 2 and Shank 3 antibodies for their target protein at the level of western blotting and immunocytochemistry using Shank overexpression and using hippocampal material from Shank2^−/−^ and Shank3αβ^−/−^ mice. In immunoblot analysis, the antibodies specifically recognize the protein at the expected molecular weight (Supplementary Figures S1B,C,E). More important for this study, immunocytochemical stainings of the utilized antibodies reveal that they specifically recognize their target protein in hippocampal sections (Supplementary Figure S1D).

### Shank1-3 exhibit a synaptic/somato-synaptic distribution in the hippocampus with a prominent immunoreactivity in the mossy fibers

**Figure 1:**
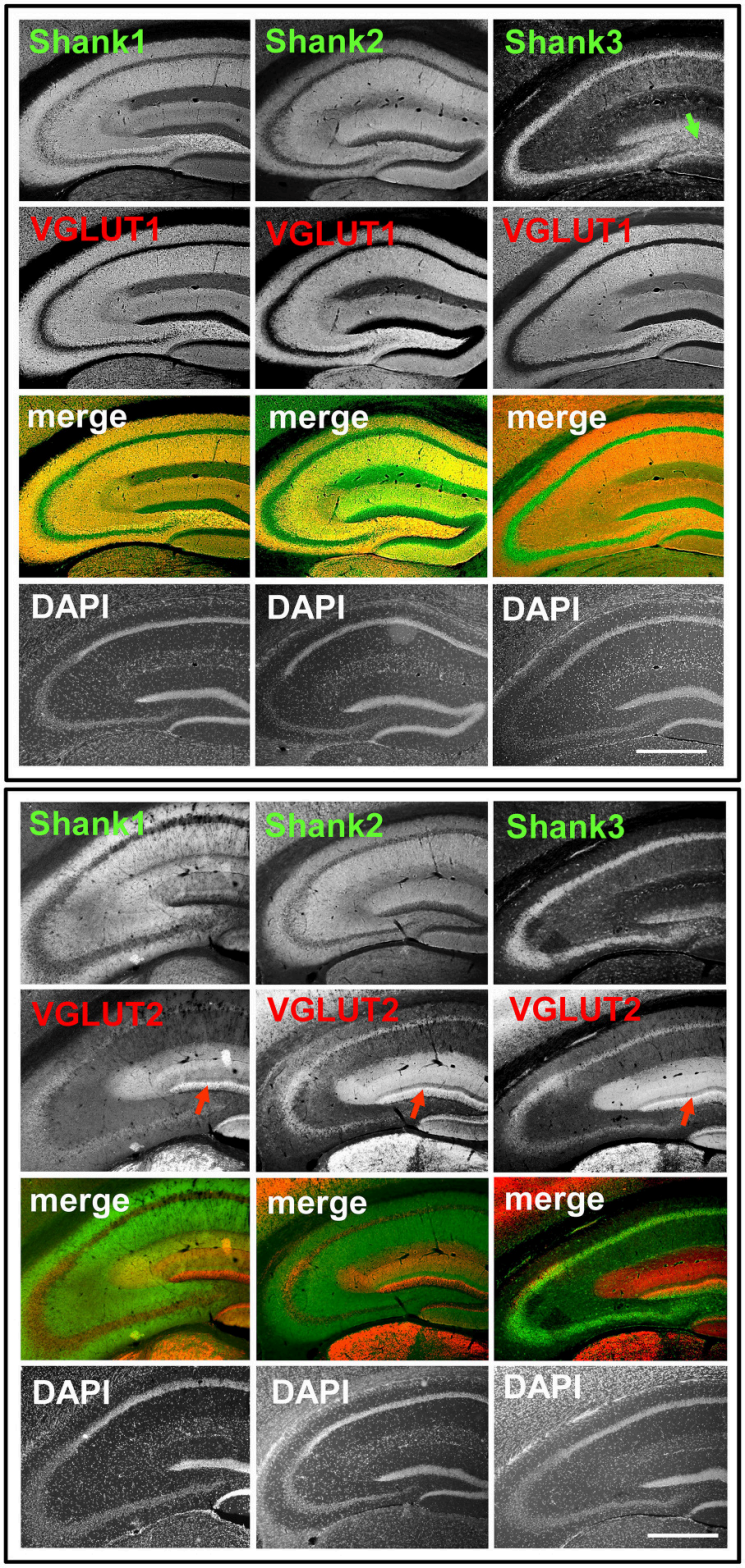
**Synaptic distribution of Shank1/2 and somato-synaptic distribution of Shank3 in the mouse hippocampus, codistribution with VGLUT1, no codistribution with VGLUT2**. **Top box**: 5x magnification of hippocampus. Immunofluorescence stainings of coronal sections from wild-type mice probed with Shank1-3 (white; green in merge) and VGLUT1 (white; red in merge) antibodies; green arrow points toward the intragranular mossy fibers where there is a prominent synaptic stain of Shank3; scale bar = 300 μm. **Bottom box**: same stainings as top box but with the VGLUT2 antibody (white; red in merge). Red arrow points toward the VGLUT2-band of the DG; scale bar = 300 μm.

**Figure 2:**
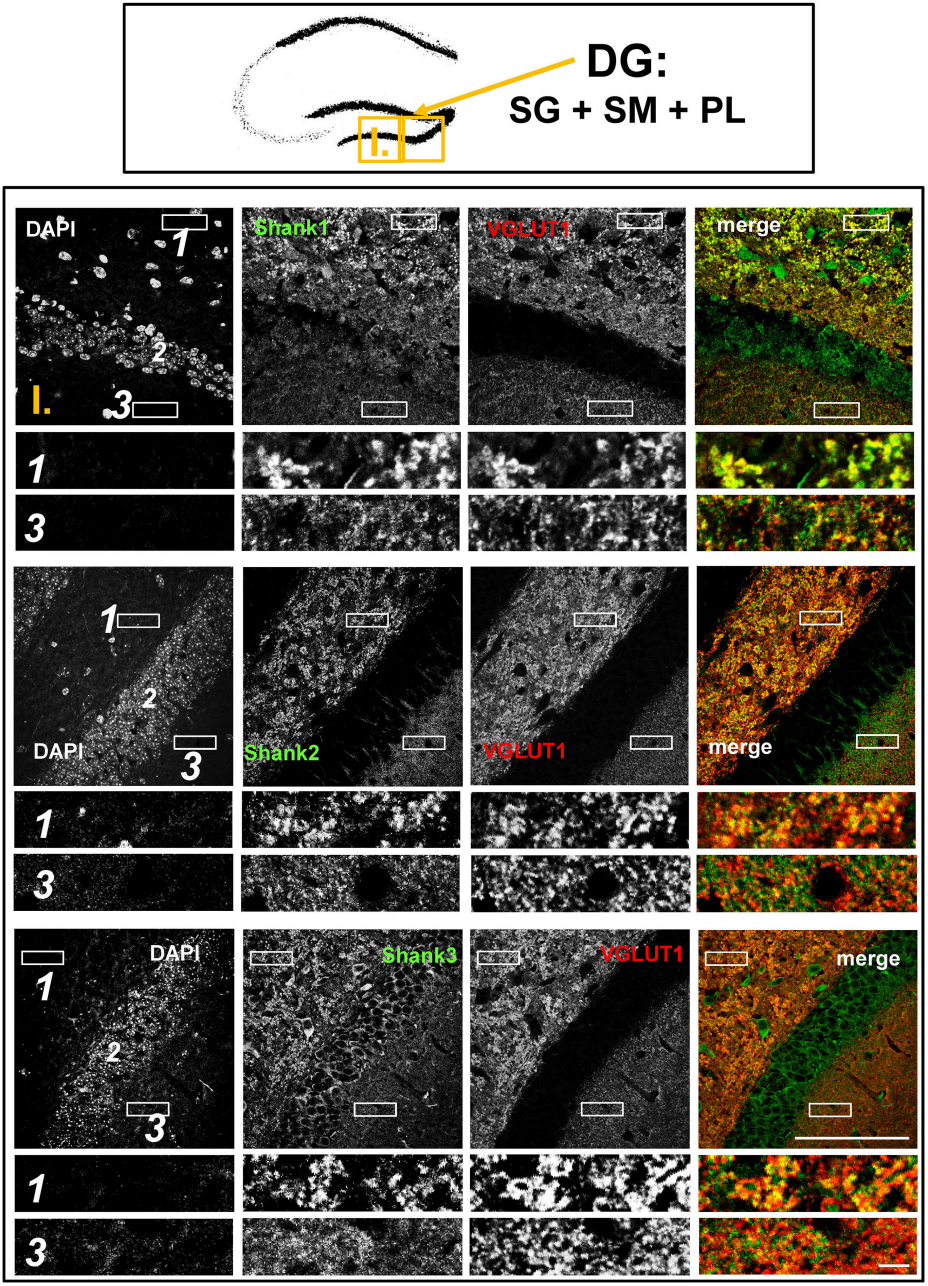
**Colocalization of Shank puncta with VGLUT1 puncta in the intragranular mossy fibers and the stratum moleculare of the dentate gyrus**. **Top box**: Schematic overview of hippocampus with enlarged region (yellow square; here: dentate Gyrus = DG, containing stratum granulare = SG, stratum moleculare = SM, and polymorphic layer = PL; I. indicates uppermost row/figure in bottom box). **Bottom box**: confocal immunofluorescence stainings of coronal sections from wild-type mice probed with the Shank1-3 (white; green in merge) and VGLUT1 (white; red in merge) antibodies. The upper rows (large squares) show the enlarged region (3 = SM; 2 = SG; 1 = PL; scale bar = 100 μm), the bottom rows (small rectangles) show further enlargements (indicated in upper row by white rectangles) in the PL (1, top row) and SM (3, bottom row) (scale bar = 5 μm).

**Figure 3:**
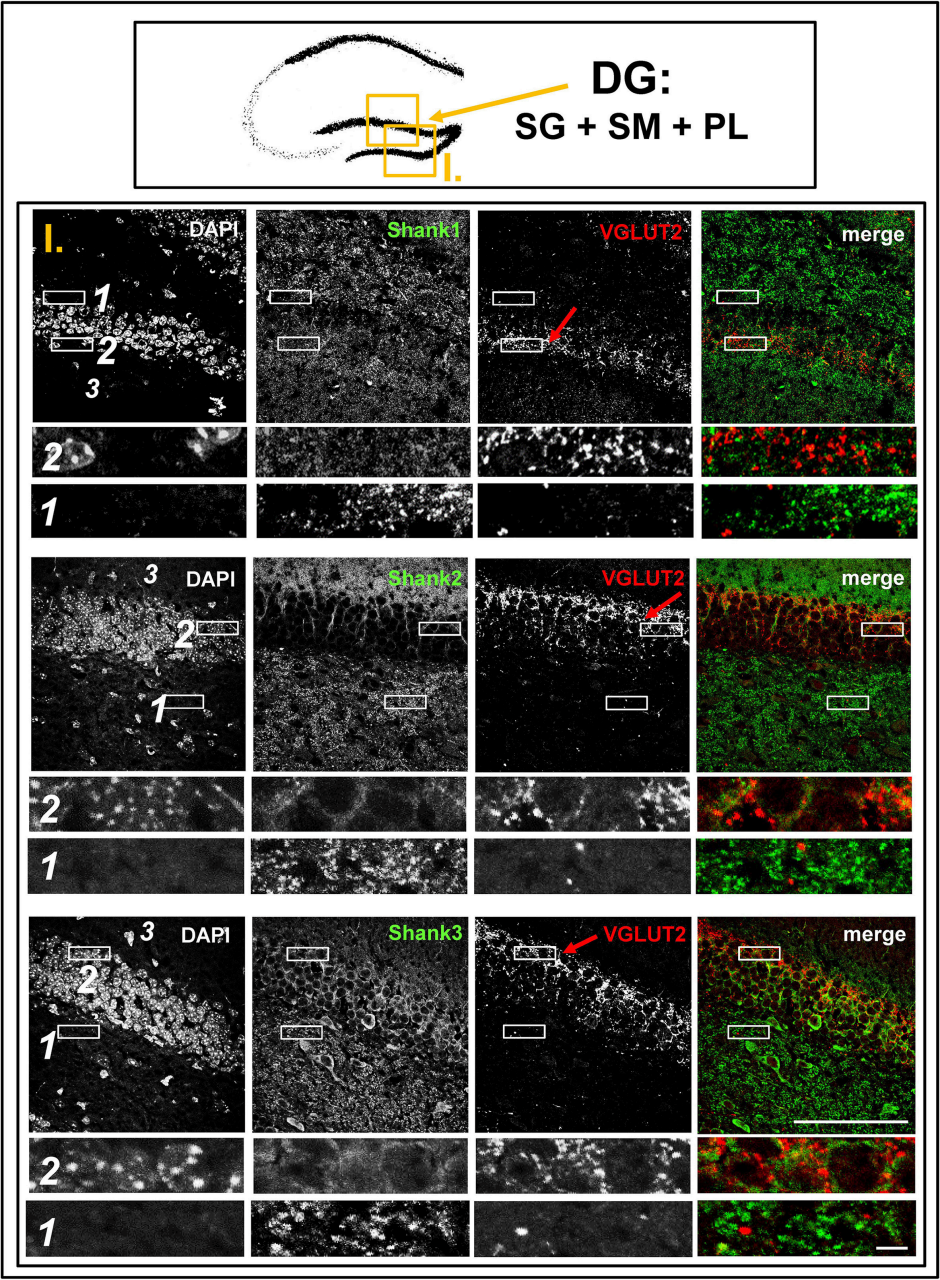
**No colocalization of Shank puncta with VGLUT2 puncta in the VGLUT2-band nor in the intragranular mossy fibers of the dentate gyrus**. **Top box**: Schematic overview of hippocampus with enlarged region (yellow square; here: dentate Gyrus = DG, containing stratum granulare = SG, stratum moleculare = SM, and polymorphic layer = PL; I. indicates uppermost row/figure in bottom box). **Bottom box**: confocal immunofluorescence stainings of coronal sections from wild-type mice probed with the Shank1-3 (white; green in merge), and VGLUT2 (white; red in merge) antibodies. The upper rows (large squares) show the enlarged region (3 = SM; 2 = SG; 1 = PL; scale bar = 100 μm), the bottom rows (small rectangles) show further enlargements (indicated in upper row by white rectangles) in the outer part of the SG (2, top row) and in the PL (1, bottom row) (scale bar = 5 μm). Red arrows indicate VGLUT2-band.

**Figure 4:**
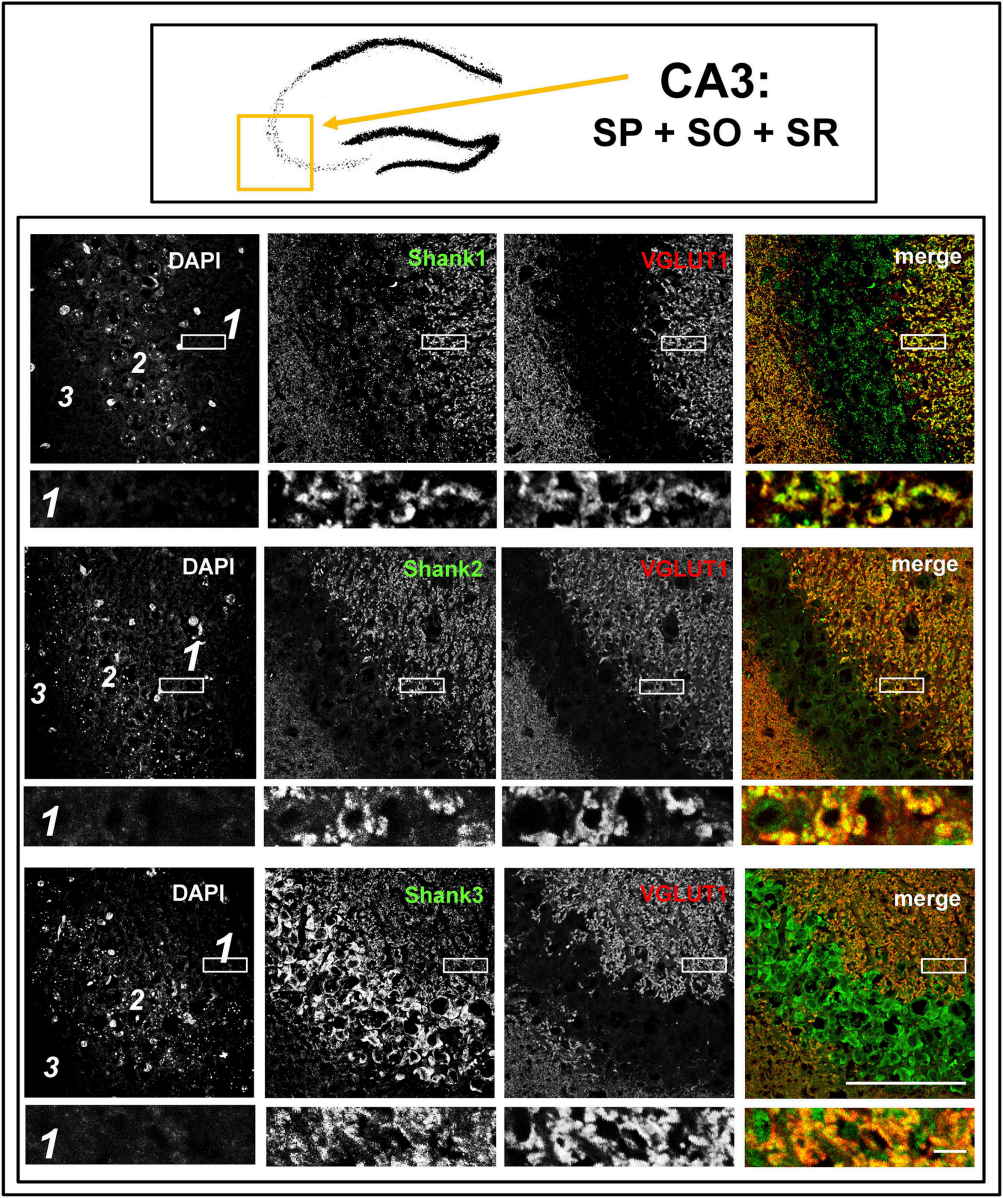
**Colocalization of Shank immunoreactivity with VGLUT1 in the stratum radiatum/stratum lucidum of the CA3 region**. **Top box**: Schematic overview of hippocampus with enlarged region (yellow square; here: CA3 containing stratum pyramidale = SP, stratum oriens = SO and stratum radiatum = SR). **Bottom box**: confocal immunofluorescence stainings of coronal sections from wild-type mice probed with the Shank1-3 (white; green in merge) and VGLUT1 (white; red in merge) antibodies. The upper row (large squares) shows the enlarged region (3 = SO; 2 = SP; 1 = SR; scale bar = 100 μm), the bottom row (small rectangles) shows a further enlargement (indicated in upper row by white rectangle) in the SR (scale bar = 5 μm).

**Figure 5:**
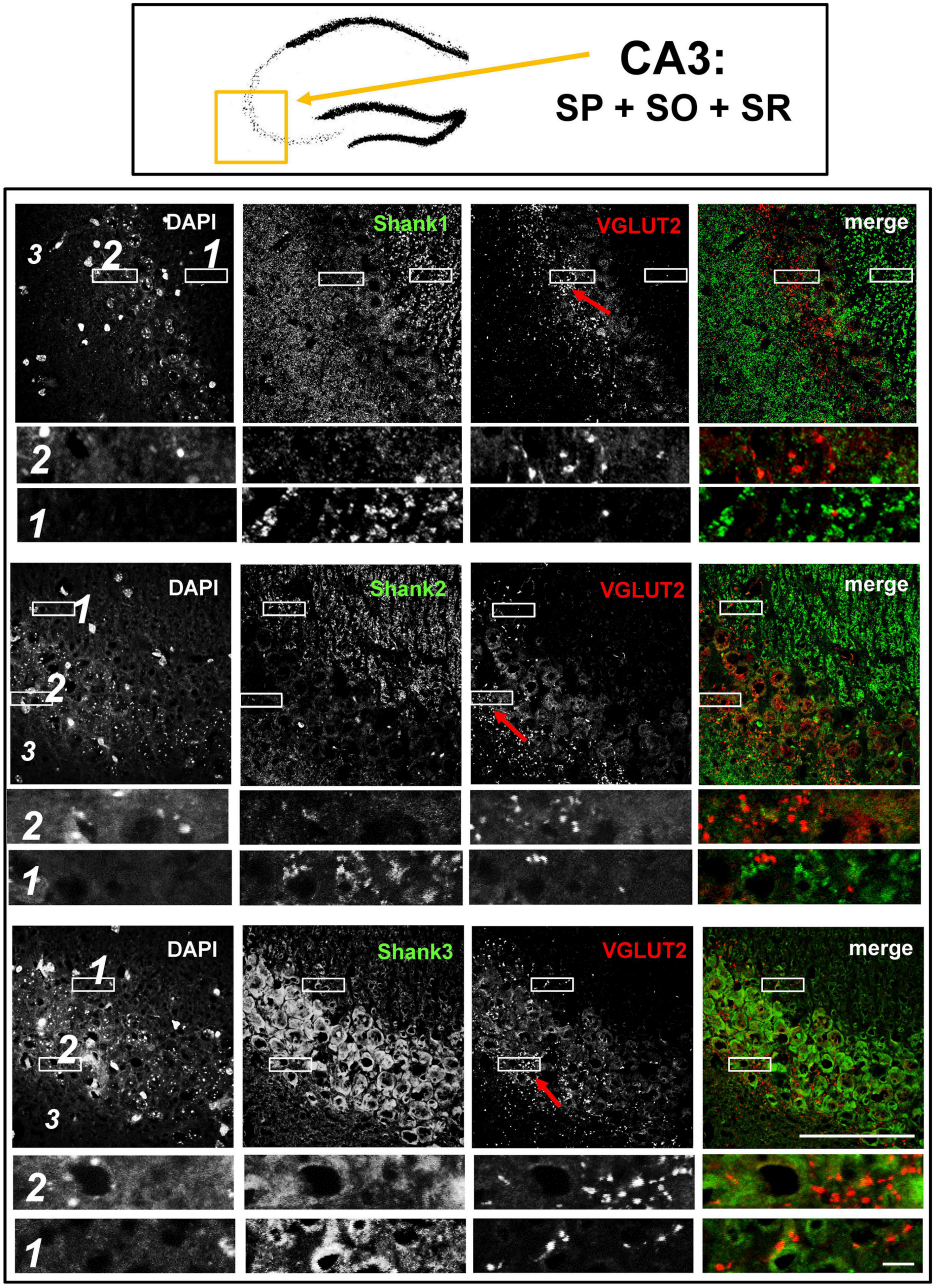
**No colocalization of Shank immunoreactivity with VGLUT2 puncta in the VGLUT2-band of the stratum pyramidale nor in the mossy fibers of the CA3**. **Top box**: Schematic overview of hippocampus with enlarged region (yellow square; here: CA3 containing stratum pyramidale = SP, stratum oriens = SO and stratum radiatum = SR). **Bottom box**: confocal immunofluorescence stainings of coronal sections from wild-type mice probed with the Shank1-3 (white; green in merge) and VGLUT2 (white; red in merge) antibodies. The upper row (large squares) shows the enlarged region (3 = SO; 2 = SP; 1 = SR; scale bar = 100 μm), the bottom row (small rectangles) shows further enlargements (indicated in upper row by white rectangles) in the lateral part of the SP (2, top row) and the SR (1, bottom row) (scale bar = 5 μm). Red arrows indicate VGLUT2-band.

We first analyzed the regional distribution of Shank1-3 in coronal sections of the mouse hippocampus. Shank1 and 2 have a similar distribution pattern as the classical excitatory synapse proteins VGLUT1 (Figure 1, top box) and GluA1 (Supplementary Figures S1, 2A) which exhibit low immunoreactivity in the somatic layers while showing strong immunoreactivity in the synaptic laminae (Balschun et al., 2010; Hagihara et al., 2011; Herzog et al., 2011). Instead, Shank3 has a more somato-synaptic distribution, exhibiting high immunoreactivity in the somatic layers while showing moderate immunoreactivity in the synaptic laminae (Figure 1). Interestingly, Shank3 shows a high immunoreactivity in the IMFs in the polymorphic layer of the DG (Figure 1, top box, green arrow; see also Figures 2, 3), which also holds true for Shank1-2 upon closer inspection using confocal microsopy (Figures 2, 3). Of note, all Shanks exhibit strong immunoreactivity not only in the IMFs but in the entire MFs, for example in the stratum radiatum/stratum lucidum of the CA3 (Figures 4, 5).

### Synaptic Shank1-3 are colocalized with VGLUT1 in the dentate Gyrus, CA3, and CA1

**Figure 6:**
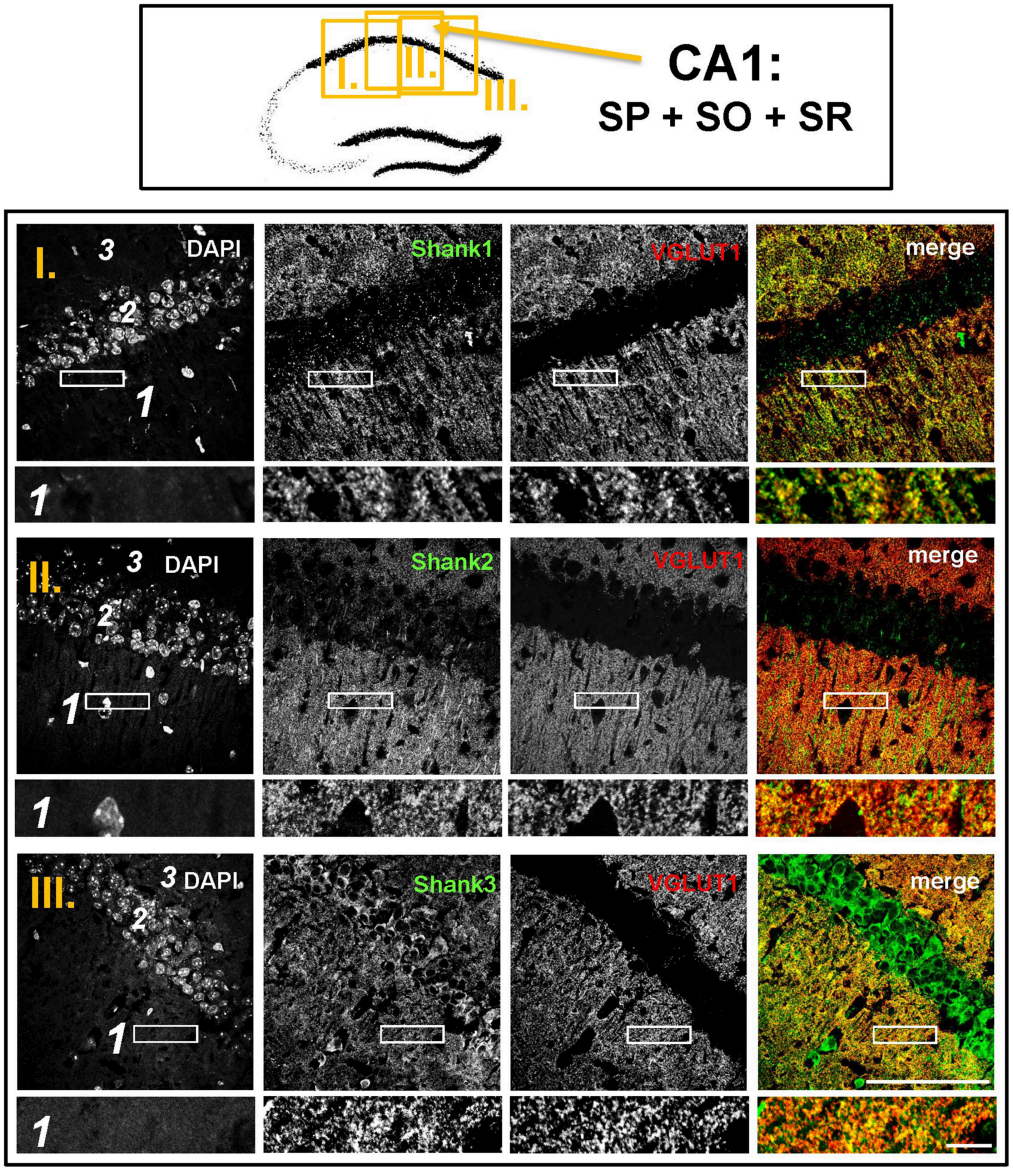
**Colocalization of Shank puncta with VGLUT1 puncta in the stratum radiatum of the CA1**. **Top box:** Schematic overview of hippocampus with enlarged region (yellow square; here: CA1 containing stratum pyramidale = SP, stratum oriens = SO and stratum radiatum = SR; I./II./III. indicates uppermost/middle/bottom figure in bottom box). **Bottom box:** confocal immunofluorescence stainings of coronal sections from wild-type mice probed with the Shank1-3 (white; green in merge) and VGLUT1 (white; red in merge) antibodies. The upper row (large squares) shows the enlarged region (3 = SO; 2 = SP; 1 = SR; scale bar = 100 μm), the bottom row (small rectangles) shows a further enlargement (indicated in upper row by white rectangle) in the SR (scale bar = 5 μm).

The global analysis of Shank1-3 immunostainings in the mouse hippocampus reveals a clear co-distribution of the synaptic Shank immunoreactivity with that of VGLUT1 (Figure 1, top box). Upon closer inspection of the hippocampal subregions DG, CA3, and CA1 using confocal microscopy it becomes apparent that the synaptic Shank immunoreactivity is punctated (except for stratum lucidum/stratum radiatum in CA3 where it takes on a larger, arched appearance) and is co-localized with VGLUT1 and GluA1 (Figures 2, 4, 6; Supplementary Figures S2, 1–3, S4). This co-localization is especially evident in the MF synapses (Figures 2, 4), which can be identified by a colocalization of VGLUT1 with Synaptoporin (SPO) (Supplementary Figures S1, 2C, S4), indicating MF boutons (Williams et al., 2011).

### Synaptic Shank1-3 do not colocalize with VGLUT2 in the dentate Gyrus or CA3

In contrast to VGLUT1, we observed that VGLUT2 (and VGLUT3) have distribution patterns in the hippocampus which do not follow that of a “classical” synaptic protein at the excitatory synapse since a strong stain can be observed in somatic laminae (Figure 1 bottom box; Supplementary Figures S1, 2B). In fact, VGLUT2 shows its strongest immunoreactivity in the outer part of the stratum granulare in the DG (Figure 1 bottom box, red arrows; Figure 3) followed by a strong immunoreactivity in the outer part of the stratum pyramidale in the CA3/CA2 (Figures 1, 5). These areas of strong VGLUT2 immunoreactivity take on the appearance of bands (henceforth referred to as “VGLUT2-bands”) and have been previously described (Halasy et al., 2004; Boulland et al., 2009). Of note, there is only a very low VGLUT2 signal in the CA1 subregion (Figure 1 bottom box) which was not detectable by confocal imaging (data not shown).

Interestingly, upon closer inspection of the DG and the CA3 with confocal microscopy, we found that Shank1-3 immunoreactivity is not colocalized with VGLUT2 (nor VGLUT3), neither in the VGLUT2-bands nor in the MFs (Figures 3,5; Supplementary Figures S3, 3, data for VGLUT3 is only shown for DG; Supplementary Figures S2, 2, S2, 3). We also costained VGLUT2 with the excitatory synapse molecule Homer1b/c, with the inhibitory synapse molecules VGAT and GAD65, and with Synapsin (Syn)1/2 and Bassoon which are both localized at the excitatory and inhibitory presynapse (Richter et al., 1999; Bragina et al., 2007). We found that VGLUT2-positive synapses are colocalized with Syn1/2, partially colocalized with Bassoon, and not colocalized with Homer 1b/c (Supplementary Figures S3, 1, S5, 1). VGLUT2 is also colocalized with VGAT and partially colocalized with GAD65 in the VGLUT2-band of the DG which also holds true for the VGLUT2-band in CA3 though to a lesser degree. Of note, VGLUT2 puncta in the MFs exhibit a lower degree of colocalization with VGAT and GAD65 than is the case in the VGLUT2-bands (Supplementary Figures S3, 2, S5, 2). VGLUT3 only shows a partial colocalization with VGAT and GAD65 and in the MFs this colocalization cannot be observed (Supplementary Figures S3, 4).

## Discussion

To date, there has been no proper analysis of the detailed synaptic distribution of Shanks in the hippocampus or other ASD-associated brain regions, which is quite surprising given the strong link between *SHANK2* and especially *SHANK3* mutations, ASD, and other neuropsychiatric disorders (Guilmatre et al., 2014; Leblond et al., 2014). Therefore, we set out to analyse the immunohistochemical localization of Shank1-3 in the mouse hippocampus using specific antibodies and confocal microscopy. Our macroscopic analysis revealed that Shank1-3 exhibit a localization in synaptic hippocampal laminae but it is important to point out that Shank3 also demonstrates a visible somatic localization, suggesting additional roles of Shank3 in neurons aside of its role as a scaffolding molecule at the PSD. Based on our confocal microscopy data, we conclude that Shanks do not uniformly reside at all excitatory postsynapses in the hippocampus but rather form the postsynaptic scaffold at all “classical,” VGLUT1-positive synapses, which is particularly visible at synapses within the MFs. Instead, Shanks do not participate in forming the postsynaptic scaffold at hippocampal VGLUT2-positive synapses- neither in the somatically localized VGLUT2-bands, nor in the MFs. This is surprising since it has been described that other molecules specific for excitatory synapses are present at these synaptic contacts and also at other VGLUT2-positive synapses in various brain regions (Boulland et al., 2009; Micheva et al., 2010; Soiza-Reilly and Commons, 2011). In line with previous work, we observed that VGLUT2 (and to a lesser degree VGLUT3) partially colocalizes with molecules of the inhibitory synapse in the somatic layers of the hippocampus (Herzog et al., 2004; Zander et al., 2010) but interestingly our data also shows that a large fraction of VGLUT2- (and especially VGLUT3-) positive synapses in the MFs are probably not classical GABAergic synapses and, yet, do not colocalize with Shanks.

**Figure 7:**
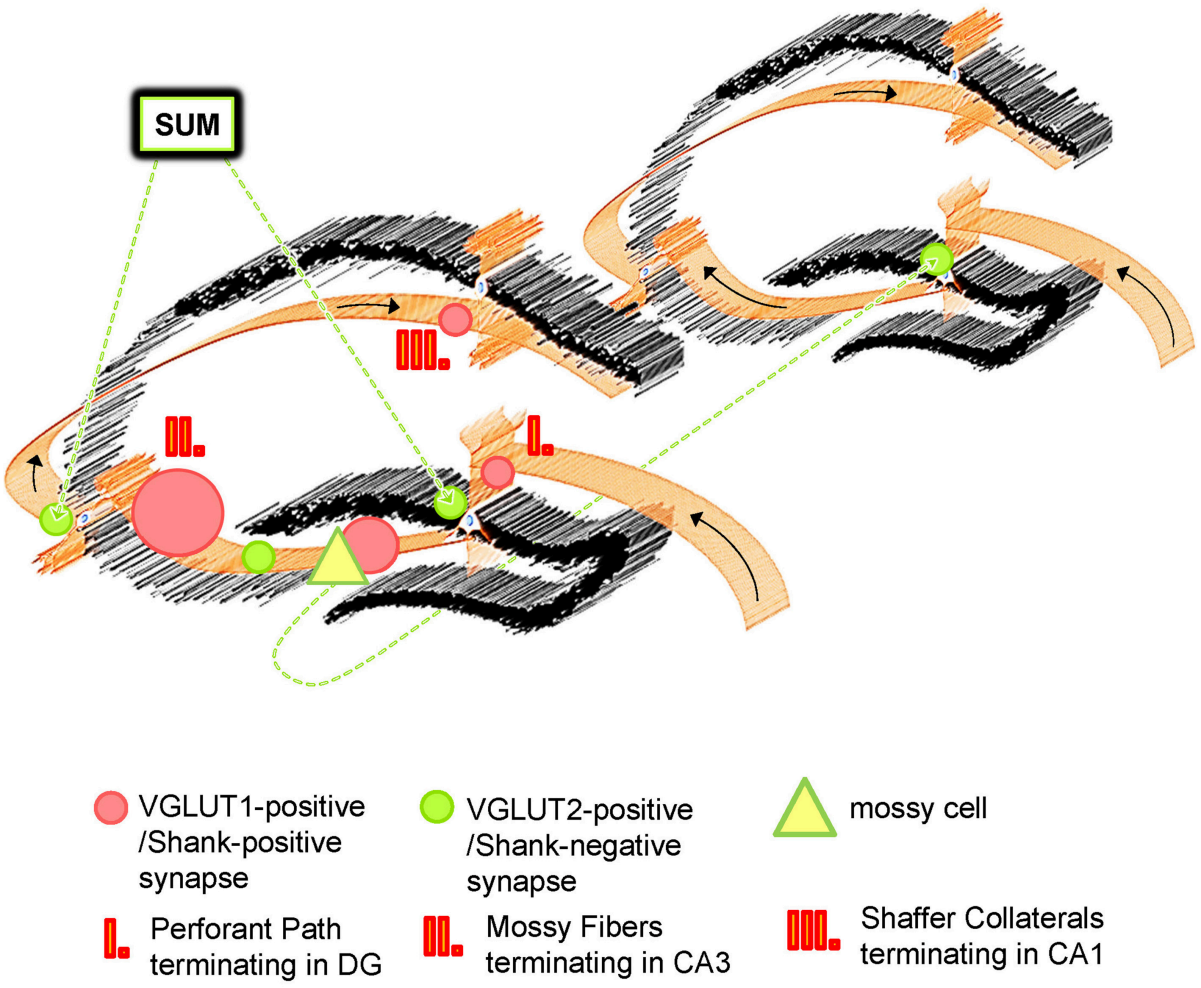
**Schematic Overview of VGLUT1/Shank-positive and VGLUT2/Shank-negative synapses in the hippocampus**. The hippocampus exhibits the well characterized trisynaptic circuit with “classical” VGLUT1-positive synapses which utilize Shank1-3, and other PSD-associated molecules at the postsynapse. Instead, VGLUT2 (and VGLUT3)-positive synapses are characterized by an absence of Shank molecules. VGLUT1- and VGLUT2-positive synapses have a complementary distribution and segregate synaptic input from different brain regions for the hippocampus. Whereas VGLUT1-positive synapses are located in non-somatic laminae throughout the hippocampus and mainly mediate input from the EC, VGLUT2-positive synapses are primarily located in the outer part of the stratum granulare of the DG and the stratum pyramidale of the CA3/CA2 as “VGLUT2-bands” and mediate input from the SUM and mossy cells. The SUM affects hippocampal function by modulating its theta rhythms, REM sleep etc. and mossy cells take part in the “crosstalk” between trisynaptic circuits along the ipsi- and contralateral extension of the hippocampus.

Interestingly, the physiology of VGLUT2-positive synapses differs from VGLUT1-positive synapses and, generally speaking, VGLUT2-positive synapses have a higher probability of transmitter release and display differences in synaptic plasticity (Fremeau et al., 2004; Boulland et al., 2009; Weston et al., 2011). Importantly, hippocampal VGLUT1- and VGLUT2-positive presynaptic terminals originate from different populations of neurons. VGLUT1-positive presynaptic terminals originate from neuronal populations within the hippocampal formation/the trisynaptic circuit (Figure 7) and from the cortex (Balschun et al., 2010; Zander et al., 2010) and are therefore heavily involved in processes like learning and memory, LTP-formation, signal processing, socio-emotional behavior, memory retrieval etc. (Shepherd, 1998; Balschun et al., 2010; Pehrs et al., 2015). Instead, VGLUT2-positive presynaptic terminals originate from the SUM or mossy cells situated along the ipsi- and contralateral extension of the hippocampus (Halasy et al., 2004; Boulland et al., 2009). Both the SUM and mossy cells have functional relevance for the hippocampus. By modulating theta rhythms, the SUM affects many processes in the hippocampus, which include learning, REM sleep, and exploratory locomotor activity (Kocsis and Vertes, 1994; Boulland et al., 2009). Mossy cells, on the other hand, are “excitatory interneurons” which receive inputs from granule cells via MF collaterals and mediate their excitation back to granule cells or basket cells along the ipsilateral extension of the hippocampus or to the contralateral hippocampal formation. Therefore, mossy cells may contribute to a “cross-talk” of trisynaptic circuits along the ipsi- and contralateral extensions of the hippocampus (Figure 7).

Given our finding that Shanks are localized to hippocampal VGLUT1- (but not VGLUT2-) positive synapses, one could expect genetic alterations of *SHANK2* or *SHANK3* (like in ASD and the Phelan-Mc-Dermid Syndrome) to have a particularly strong effect on processes involving the trisynaptic circuit if genetic buffering is insufficient (Bourgeron, 2015). For example, learning and memory, memory retrieval, or the proper exchange of information between the cortex and the hippocampus may be disrupted, possibly contributing to the abnormal socio-emotional behavior observed in ASD patients. Instead, one may expect genetic alterations of *SHANK2* or *SHANK3* to have less of an effect on VGLUT2-associated circuits in the hippocampus and, therefore, shankopathies may have a smaller impact on hippocampal theta rhythms, REM sleep, and so forth. From this perspective our findings may be viewed as part of an effort to better understand the genetic underpinnings of neuropsychiatric disorders but our investigation also contributes to the growing knowledge that there is a large degree of heterogeneity among excitatory synaptic contacts in the CNS in general (Rao et al., 1998; O'Rourke et al., 2012).

## Author contributions

CH carried out the majority of experiments and literature research, wrote the manuscript, carried out the data analysis and created the figures. JS was also involved in carrying out the experiments and helped in image acquisitions and literature research. MJS provided insight into the Shank and autism literature, revised the text, and assisted in the data analysis. SH, DR, and SW were involved in testing the specificity of Shank antibodies and provided technical support. MJS helped designing the experiments, revised the text, and provided expertise for the scientific background of the project. MK and TB designed the experiments, revised the text, and provided expertise for the project.

## Funding

The research leading to these results has received funding from the People Programme (Marie Curie Actions) of the European Union's Seventh Framework Programme FP7/2007-2013/ under REA grant agreement n° 289581 and received support from the Innovative Medicines Initiative Joint Undertaking under grant agreement n° 115300, resources of which are composed of financial contribution from the European Union's Seventh Framework Programme (FP7/2007-2013) and EFPIA companies' in kind contribution (EU-AIMS). MS is supported by the Care-for-Rare Foundation and the Eliteprogramm of the Baden-Wuerttemberg Stiftung. MRK is supported by the Deutsche Forschungsgemeinschaft (KR1879 6-1, 7-1 SFB 779 TPB8), Bundesministerium für Forschung und Technologie (BMBF/Energi) and the Leibniz Foundation (Pakt für Forschung). TB is supported by grants from the Deutsche Forschungsgemeinschaft (DFG: BO 1718/4-1 and SFB1149, TPA02) and by the Virtual Institute within the Helmholtz Gesellschaft (“RNA Dysmetabolism in ALS and FTD,” VH-VI-510).

### Conflict of interest statement

The authors declare that the research was conducted in the absence of any commercial or financial relationships that could be construed as a potential conflict of interest.

## Abbreviations

ASD: autism spectrum disorders
CA1-4: Cornu Ammonis areas 1-4
DAPI: 4′6-diamidino-2-phenylindole
DG: dentate gyrus
EC: entorhinal cortex
IMF: intragranular mossy fibers
MAP2: microtubule-associated protein 2
MF: mossy fibers
PBS: phosphate buffered saline
ProSAP: proline-rich synapse-associated protein, also known as Shank
PSD: postsynaptic density
Shank: SH3 and multiple ankyrin repeat domains 3, also known as ProSAP
SPO: Synaptoporin also known as Synaptophysin 2
SUM: supramammilary nucleus
Syn1/2: Synapsin1/2
VGLUT: vesicular glutamate transport.
