# Supplementary material for: Selective Localization of Shanks to VGLUT1-Positive Excitatory Synapses in the Mouse Hippocampus
Source: Front Cell Neurosci. 2016 Apr 26;10:106. doi: 10.3389/fncel.2016.00106 (PMC4844616; doi:10.3389/fncel.2016.00106)
Supplement: Supplementary file 1 [file DataSheet1.PDF]

Supplementary Material

Selective localization of Shanks to VGLUT1-positive excitatory synapses in the mouse hippocampus

Christopher Heise[1,2], Jan C. Schroeder[1], Michael Schoen[1], Sonja Halbedl[1], Dominik Reim[1], Sarah Woelfle[1,] Michael R. Kreutz[2], Michael J. Schmeisser[1,3] and Tobias M. Boeckers[1*]

[*] **Correspondence:** Tobias M. Boeckers, Institute of Anatomy and Cell Biology, Ulm University, Albert-Einstein-Allee 11, Ulm, 89073, Germany, tobias.boeckers@uni-ulm.de

Supplementary Figures

Supplementary Figure S1, 1

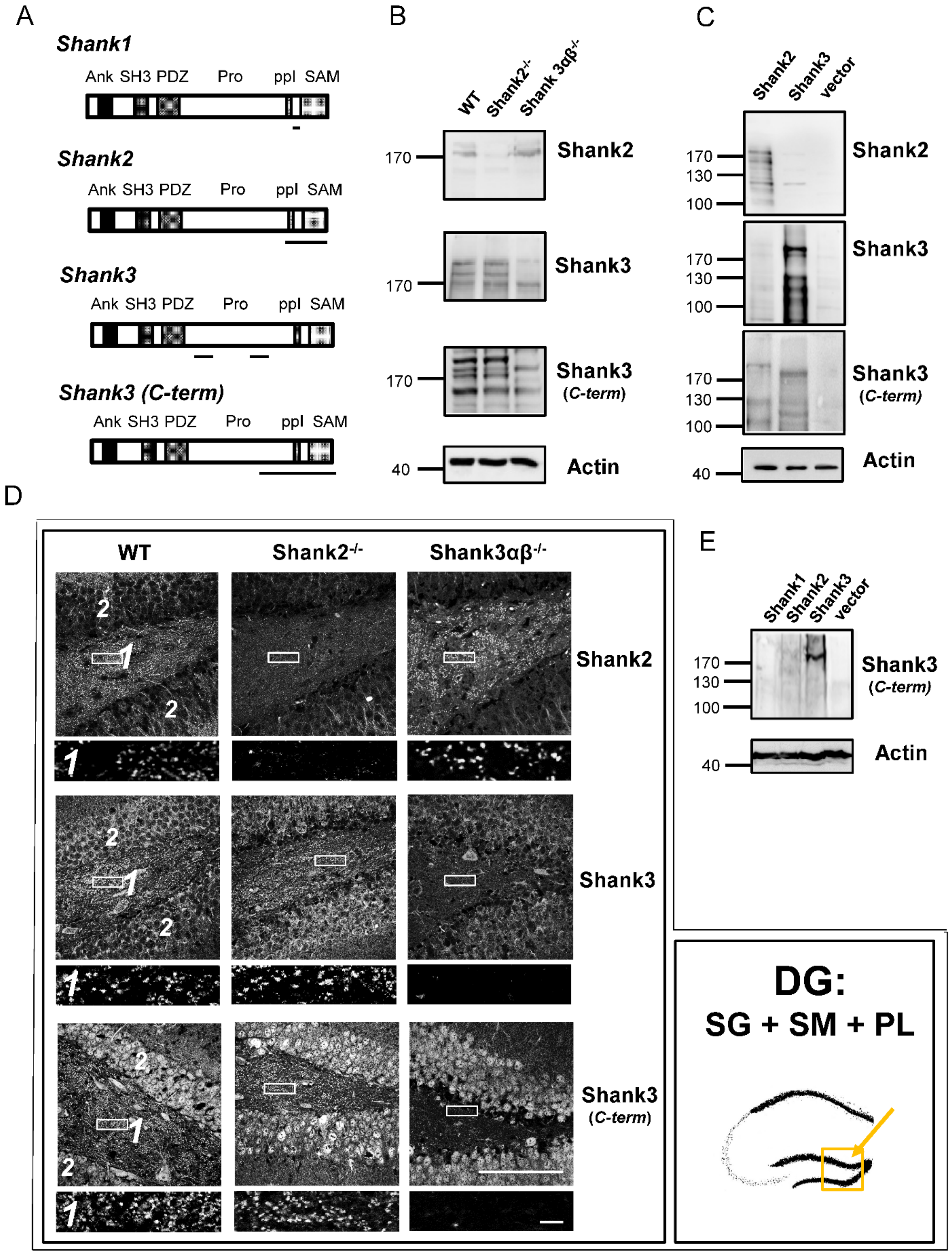

Supplementary Figure S1, 1: High specificity of used Shank antibodies

A, Schematic overview of Shank domains with epitope regions of Shank antibodies. Ank = ankyrin repeat domain; SH3 = Src homology 3 domain; PDZ = PSD-95/Discs large/ZO-1 domain; Pro = a proline-rich region containing homer- and cortactin-binding sites; ppI = cortactin binding domain; SAM = sterile alpha motif domain; black line = epitope region. **B,** Western blot of wild-type (WT), Shank2$^{-/-}$, and Shank3αβ$^{-/-}$ crude synaptosomal hippocampal fraction. Detection with Shank2, Shank3, and Shank3 („C-term“) antibodies. Residual bands in Shank3αβ$^{-/-}$ have been previously described (Schmeisser et al., 2012). **C,** Western blot of HEK cell lysates with human Shank2-3 overexpression or **E,** rat Shank1-3 overexpression. Detection with Shank2, Shank3, and Shank3 („C-term“) antibodies. **D, bottom right box**: Schematic overview of hippocampus with enlarged region (yellow square; here: Dentate Gyrus = DG, containing stratum granulare = SG, stratum moleculare = SM, polymorphic layer = PL). **D, left box**: confocal immunofluorescence stainings of coronal sections from wild-type (WT), Shank2$^{-/-}$, and Shank3αβ$^{-/-}$ mice probed with the Shank2, Shank3, and Shank3 („C-term“) antibodies (white). The upper rows (large squares) show the enlarged region (2 = SG; 1 = PL; scale bar = 100 µm), the bottom rows (small rectangles) show further enlargements (indicated in upper row by white rectangle) in the PL, a region with prominent synaptic Shank stain (scale bar = 5 µm).

Supplementary Figure S1, 2:

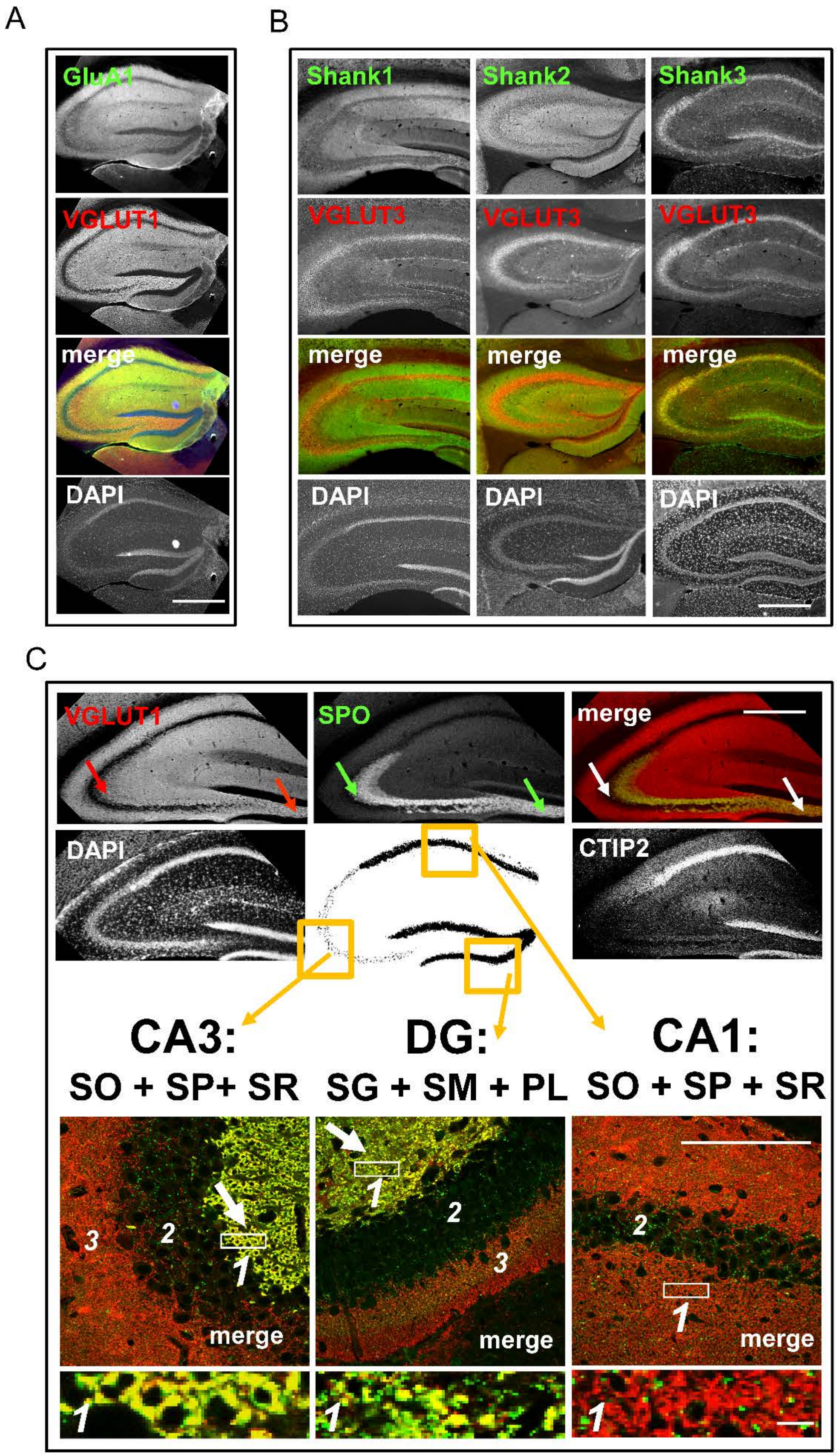

Supplementary Figure S1, 2: Codistribution of VGLUT1 with GluA1, colocalization of VGLUT1 with SPO in the mossy fibers, and somato-synaptic distribution of VGLUT3 in the mouse hippocampus.

A, 5x magnification of hippocampus. Immunofluorescence stainings of coronal sections from wild-type mice probed with the GluA1 (white; green in merge) and VGLUT1 antibodies (red; green in merge) . Scale bar (white line) = 300 µm. **B,** 5x magnification of hippocampus. Immunofluorescence stainings of coronal sections from wild-type mice probed with Shank1-3 (white; green in merge) and VGLUT3 (white; red in merge) antibodies; scale bar (white line) = 300 µm.**C, top** 5x magnification of hippocampus. Immunofluorescence stainings of coronal sections from wild-type mice probed with the VGLUT1 (red; green in merge) , Synaptophysin2/SPO (white; green in merge) , and CTIP2 antibodies (white). Red/green/white arrows point towards DG (right) and the CA3 region (left), where VGLUT1 and SPO colocalize, indicating mossy fiber boutons; scale bar (white line) = 300 µm. **C, middle:** Schematic overview of hippocampus with enlarged regions (yellow squares; here: CA3 containing stratum pyramidale = SP, stratum oriens = SO and stratum radiatum = SR; Dentate Gyrus = DG, containing stratum granulare = SG, stratum moleculare = SM, and polymorphic layer = PL; CA1 containing stratum pyramidale = SP, stratum oriens = SO and stratum radiatum = SR). **C, bottom:** confocal immunofluorescence stainings of coronal sections from wild-type mice probed with the VGLUT1 (red in merge) and SPO antibodies (green in merge). The upper row shows the enlarged region (3 = SO/SM 2 = SP/SG; 1 = SO/PL; scale bar = 100 µm), the bottom row shows further enlargements (indicated in top row by white rectangle) in the SR/stratum lucidum of the CA3 (left), PL of the DG (middle), and SR of the CA1 (right) (scale bar = 5 µm). White arrows indicate mossy fibers boutons in the CA3 and DG which are characterized by a colocalization of VGLUT1 and SPO.

Supplementary Figure S2, 1

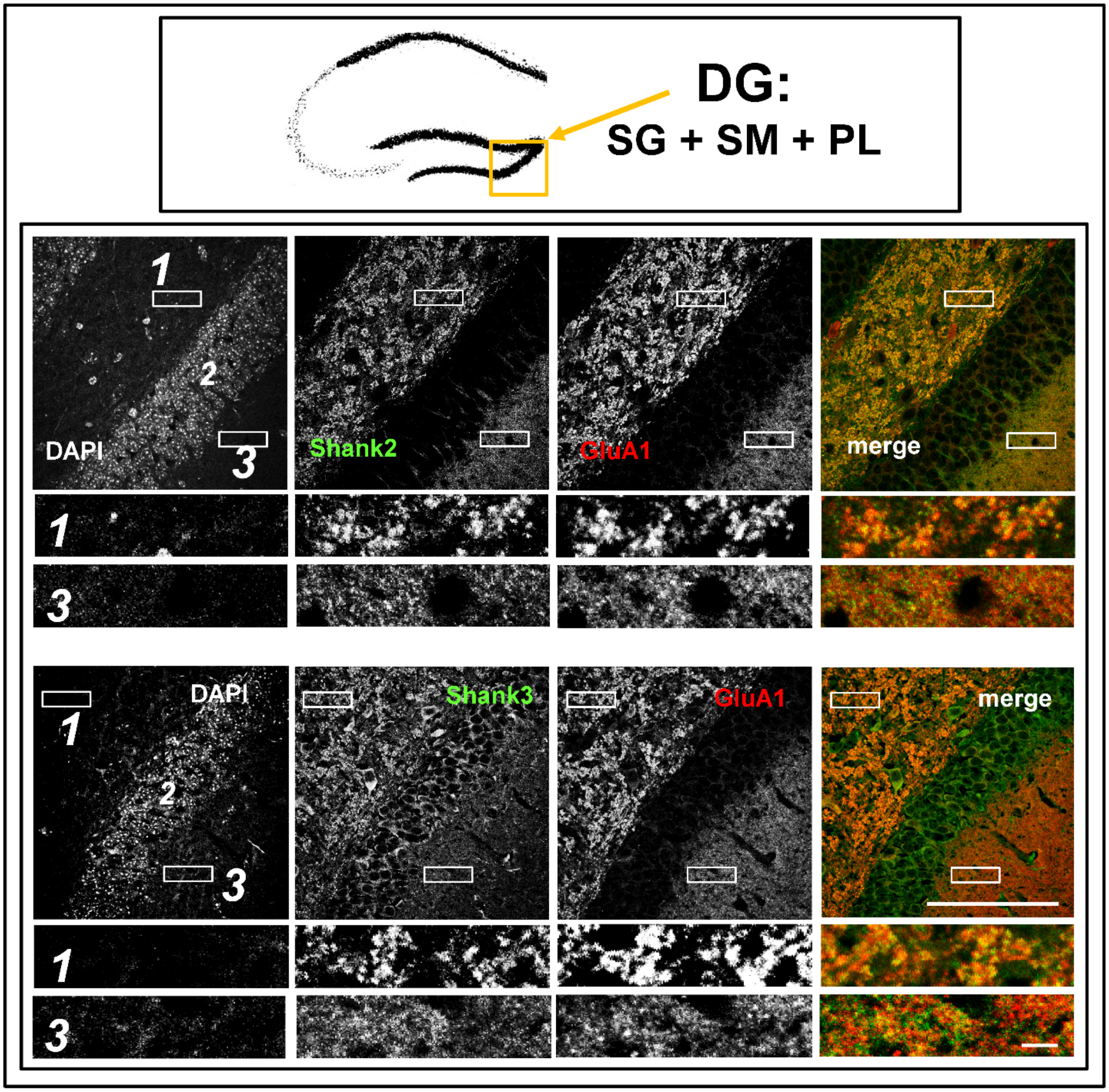

Supplementary Figure S2, 1: Colocalization of Shank2 and Shank3 puncta with GluA1 puncta in the stratum moleculare and the intragranular mossy fibers of the dentate gyrus.

Top box: Schematic overview of hippocampus with enlarged region (yellow square; here: dentate Gyrus = DG, containing stratum granulare = SG, stratum moleculare = SM, and polymorphic layer = PL). **Bottom box:** confocal immunofluorescence stainings of coronal sections from wild-type mice probed with the Shank2 (white; green in merge) or Shank3 (white; green in merge), and GluA1 (white; red in merge) antibodies. The upper rows (large squares) show the enlarged region (3 = SM; 2 = SG; 1 = PL; scale bar = 100 µm), the bottom rows (small rectangles) show further enlargements (indicated in upper row by white rectangles) in the PL (1, top row) and SM (3, bottom row) (scale bar = 5 µm).

Supplementary Figure 2, 2

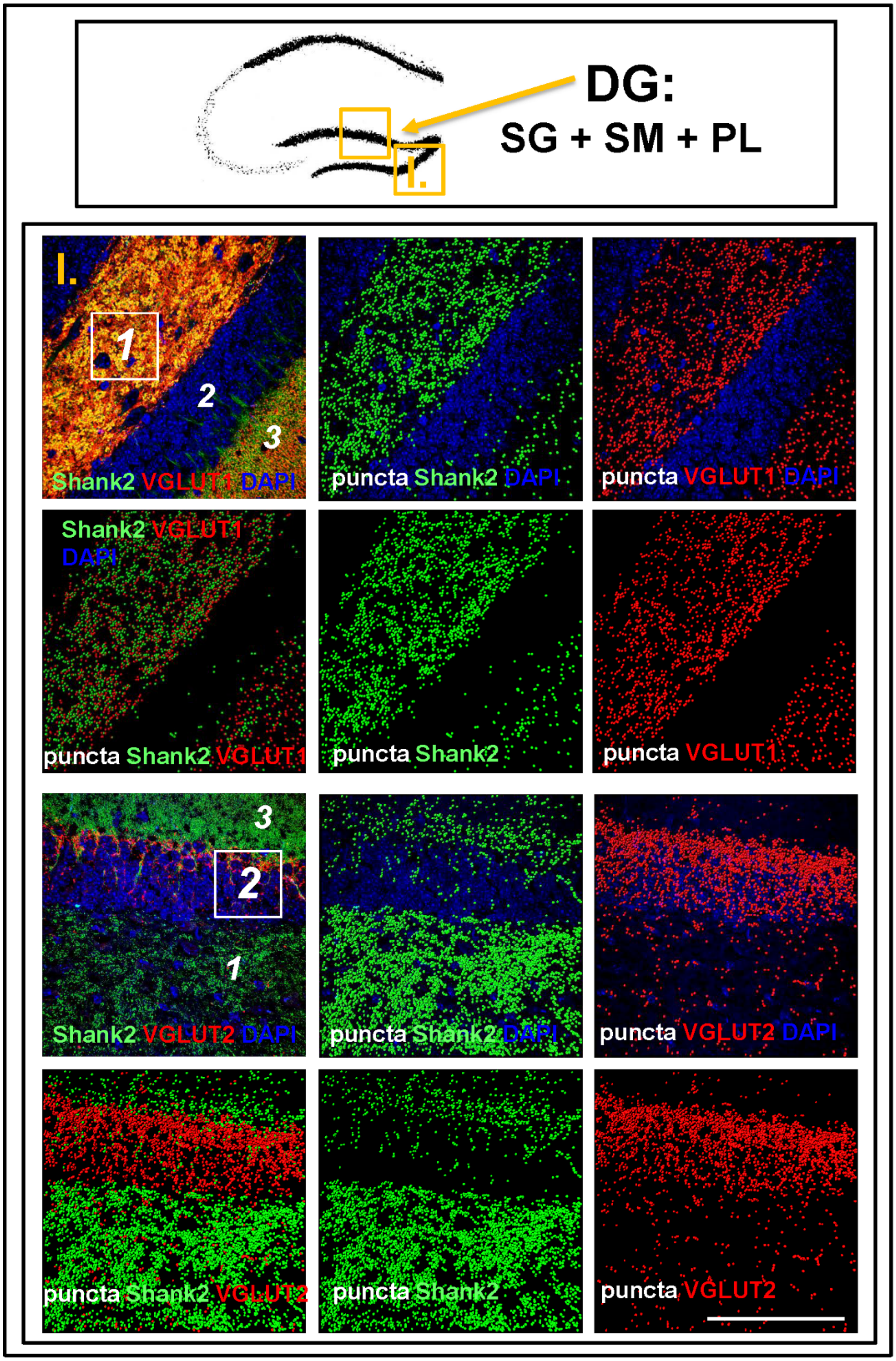

Supplementary Figure 2, 2: Approach of quantitative puncta analysis of Shanks and VGLUTs using Imaris, exemplified by representative Shank2 and VGLUT1/2 stains in the dentate gyrus of the mouse hippocampus. **Top box:** Schematic overview of hippocampus with enlarged region (yellow square; here: dentate Gyrus = DG, containing stratum granulare = SG, stratum moleculare = SM, and polymorphic layer = PL; I. indicates upper two rows in bottom box). **Bottom box:** Puncta analysis of confocal immunofluorescence stainings of coronal sections from wild-type mice probed with the Shank2 (green), and VGLUT2 (red) antibodies. The top left image of the upper two rows shows a representative Shank2 and VGLUT1 stain, the top left image of the bottom two rows shows a representative Shank2 and VGLUT2 stain (3 = SM; 2 = SG; 1 = PL; white rectangles indicate regions that were selected for subanalysis, view Supplementary Figure 2, 3). To the right of the actual images are a series of corresponding puncta representations (Shank2 in green, VGLUT2 in red). Scale bar = 100 µm.

Supplementary Figure 2, 3

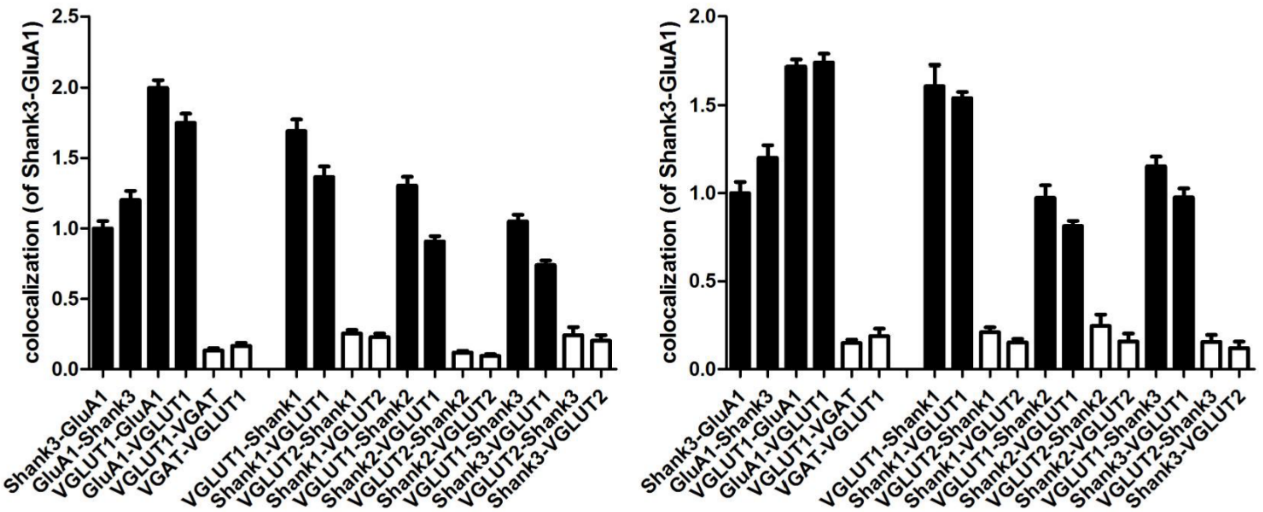

Supplementary Figure 2, 3: Quantification of Shank1-3/VGLUT puncta analysis in the dentate gyrus of the mouse hippocampus using Imaris reveals colocalization of Shanks with VGLUT1 but not with VGLUT2. Colocalization analysis is carried out for pairs of VGLUT1/2 and Shank1-3 but also with pairs of VGLUT1 and GluA1 or VGAT, as well as Shank3 and GluA1 as controls/references (GluA1 coloclalizes with Shank3 and VGLUT1; VGLUT1 does not colocalize with VGAT). Colocalization analysis is carried out with a reference mask of VGLUTs (e.g. VGLUT1-Shank1), Shanks (e.g. Shank1-VGLUT1), GluA1 (e.g. GluA1-Shank3), or VGAT (VGAT-VGLUT1). The center of mass of the identified puncta have a maximal distance of 0.7 µm. Data is normalized to the colocalization pair Shank3-GluA1. Colocalizations pertaining to VGLUT2 or VGAT are displayed in white, the other data bars are displayed in black. Analysis is based on n = 5-8 images from 3-5 different animals. The visible difference between black and white bars can also be

shown by statistical analysis (ANOVA with post hoc tukey test reveals a highly significant F-value of $p < 0.001$ and post hoc tukey test reveals highly significant differences of means with $p < 0.001$). **Left graph:** Colocalization analysis based on the entire 63x image (192.8 µm x 192.8 µm) as shown in Supplementary Figure 2, 2. **Right graph:** Colocalization analysis based on a subfield (48.2 µm x 48.2 µm) of the 63x image as shown in Supplementary Figure 2, 2 (small white squares refer to subfield). For VGLUT1 the subfield was chosen in the polymorphic layer, for VGLUT2 and VGAT the subfield was chosen in the stratum granulare.

Supplementary Figure S3, 1

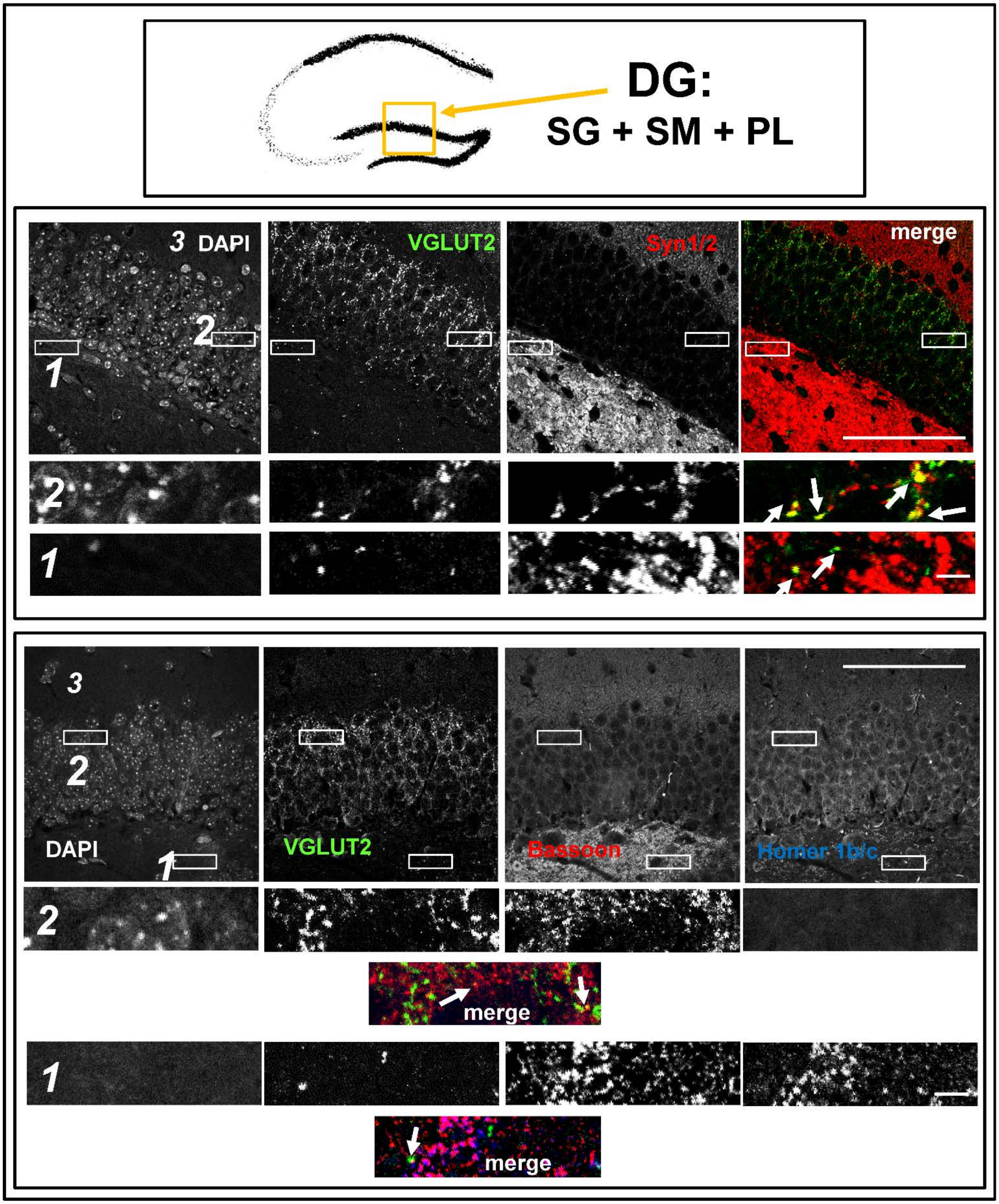

Supplementary Figure S3, 1: Colocalization of VGLUT2 and Syn1/2 puncta in the VGLUT2-band of the stratum granulare and in the intragranular mossy fibers of the dentate gyrus, low colocalization with Bassoon, no colocalization of VGLUT2 with Homer 1b/c puncta.

Top box: Schematic overview of hippocampus with enlarged regions (yellow squares; here: dentate Gyrus = DG, containing stratum granulare = SG, stratum moleculare = SM, and polymorphic layer = PL). **Middle box:** confocal immunofluorescence stainings of coronal sections from wild-type mice probed with VGLUT2 (white; green in merge) and Syn1/2 (white; red in merge) antibodies. The upper row (large squares) shows the enlarged region (3 = SM; 2 = SG; 1 = PL; scale bar = 100 µm), the bottom rows (small rectangles) show further enlargements (indicated in upper row by white rectangles) in the SG (2, top row) and PL (1, bottom row) (scale bar = 5 µm). White arrows indicate visible colocalization of VGLUT2 and Syn1/2. **Bottom box:** confocal immunofluorescence stainings of coronal sections from wild-type mice probed with the VGLUT2 (white; green in merge), Bassoon (white; red in merge) , and Homer 1b/c (white; blue in merge) antibodies. The upper row (large squares) shows the enlarged region (3 = SM; 2 = SG; 1 = PL; scale bar = 100 µm), the bottom rows (small rectangles) show further enlargements (indicated in upper row by white rectangles) in the SG (2, top row) and PL (1, bottom row) (scale bar = 5 µm). White arrows indicate selected puncta with visible colocalization of VGLUT2 and Bassoon.

Supplementary Figure S3, 2

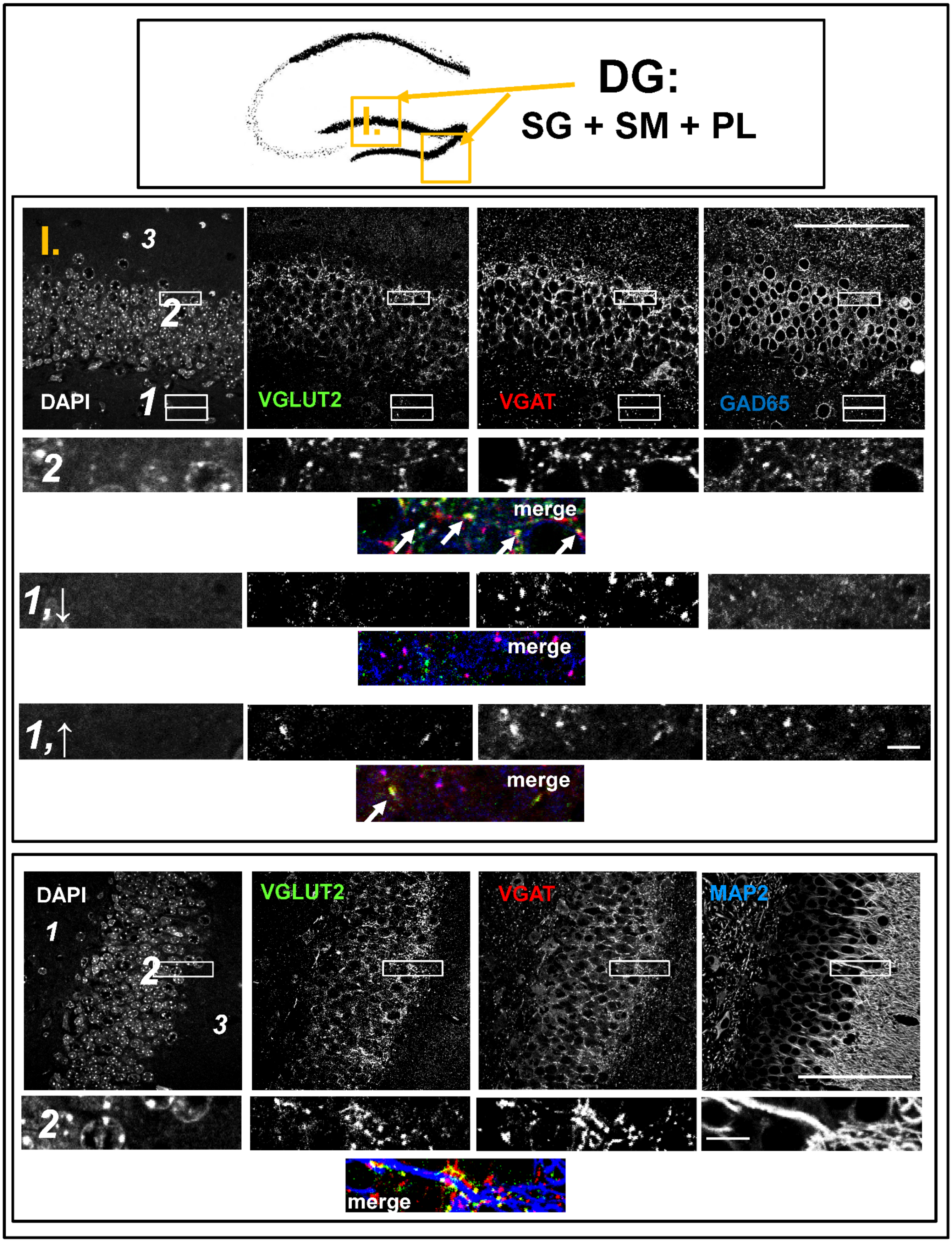

Supplementary Figure S3, 2: Colocalization of VGLUT2, VGAT, and GAD65 puncta in the VGLUT2-band of the stratum granulare but only partial colocalization in the intragranular mossy fibers synapses of the dentate gyrus.

Top box, Schematic overview of hippocampus with enlarged region (yellow square; here: dentate Gyrus = DG, containing stratum granulare = SG, stratum moleculare = SM, and polymorphic layer =PL; I. indicates uppermost row/figure in bottom box). **Middle box,** confocal immunofluorescence stainings of coronal sections from wild-type mice probed with the VGLUT2 (white; green in merge), VGAT (white; red in merge), and GAD65 (white; blue in merge) antibodies. The upper row (large squares) shows the enlarged region (3 = SM; 2 = SG; 1 = PL; scale bar = 100 µm), the bottom rows (small rectangles) show further enlargements (indicated in upper row by white rectangles) in the SG (2, top row) and PL (1↑, 1↓, middle and bottom row) (scale bar = 5 µm). White arrows indicate selected puncta with visible colocalization of VGLUT2 and VGAT, and partial colocalization with GAD65. **Bottom box,** confocal immunofluorescence stainings of coronal sections from wild-type mice probed with VGLUT2 (white; green in merge), VGAT (white; red in merge) and MAP2 (white; blue in merge) antibodies. The upper row (large squares) shows the enlarged region (3 = SM; 2 = SG; 1 = PL; scale bar = 100 µm), the bottom rows (small rectangles) show further enlargements (indicated in upper row by white rectangles) in the VGLUT-2 band in the SG (2, bottom row) (scale bar = 5 µm).

Supplementary Figure S3, 3

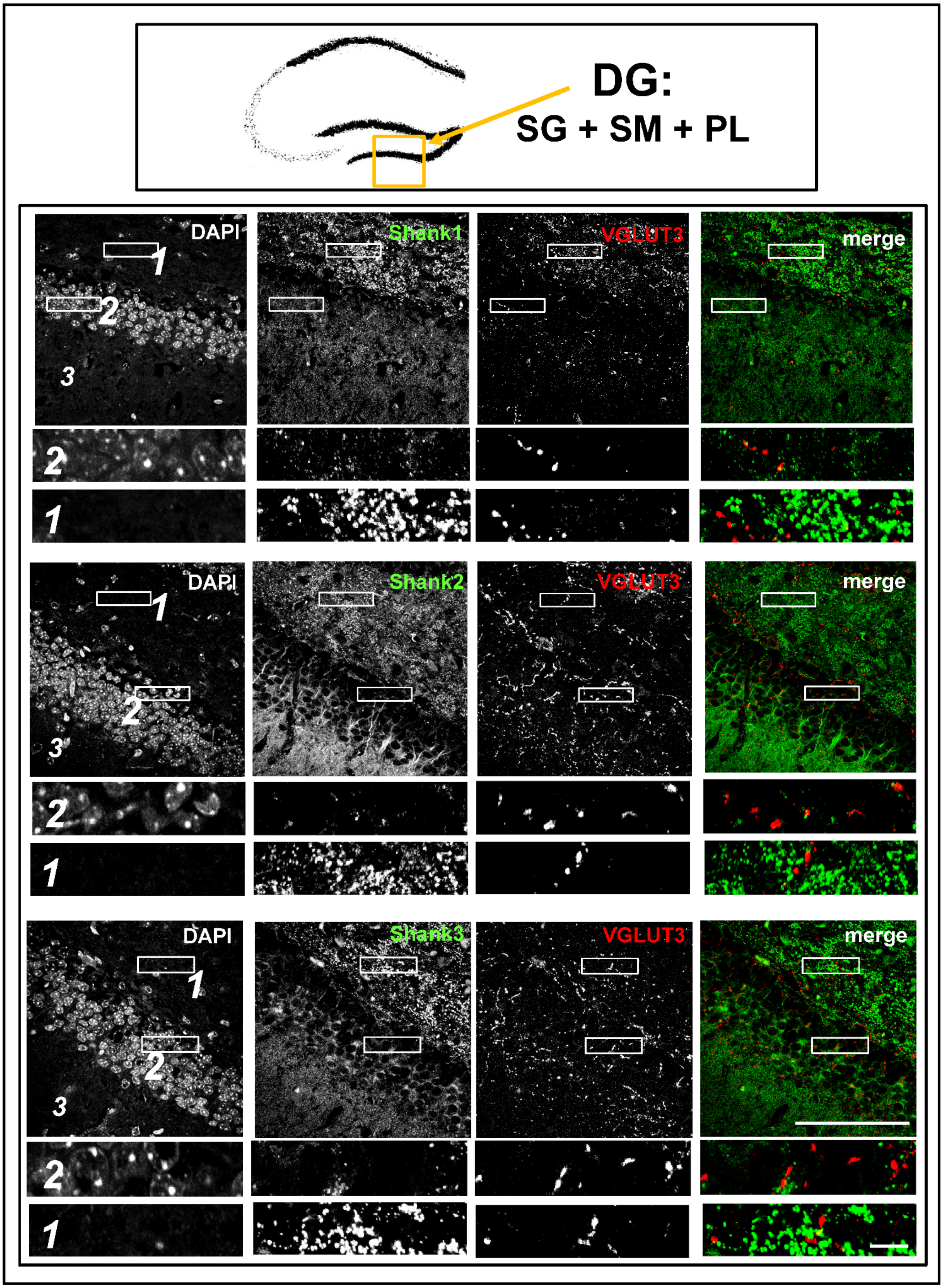

Supplementary Figure S3, 3: No colocalization of Shank puncta with VGLUT3 puncta in the stratum granulare nor in the intragranular mossy fibers of the dentate gyrus.
Top box: Schematic overview of hippocampus with enlarged region (yellow square; here: dentate Gyrus = DG, containing stratum granulare = SG, stratum moleculare = SM, and polymorphic layer = PL). **Bottom box:** confocal immunofluorescence stainings of coronal sections from wild-type mice probed with the Shank1-3 (white; green in merge), and VGLUT3 (white; red in merge) antibodies. The upper rows (large squares) show the enlarged region (3 = SM; 2 = SG; 1 = PL; scale bar = 100 µm), the bottom rows (small rectangles) show further enlargements (indicated in upper row by white rectangles) in the outer part of the SG (2, top row) and in the PL (1, bottom row) (scale bar = 5 µm).

Supplementary Figure S3, 4

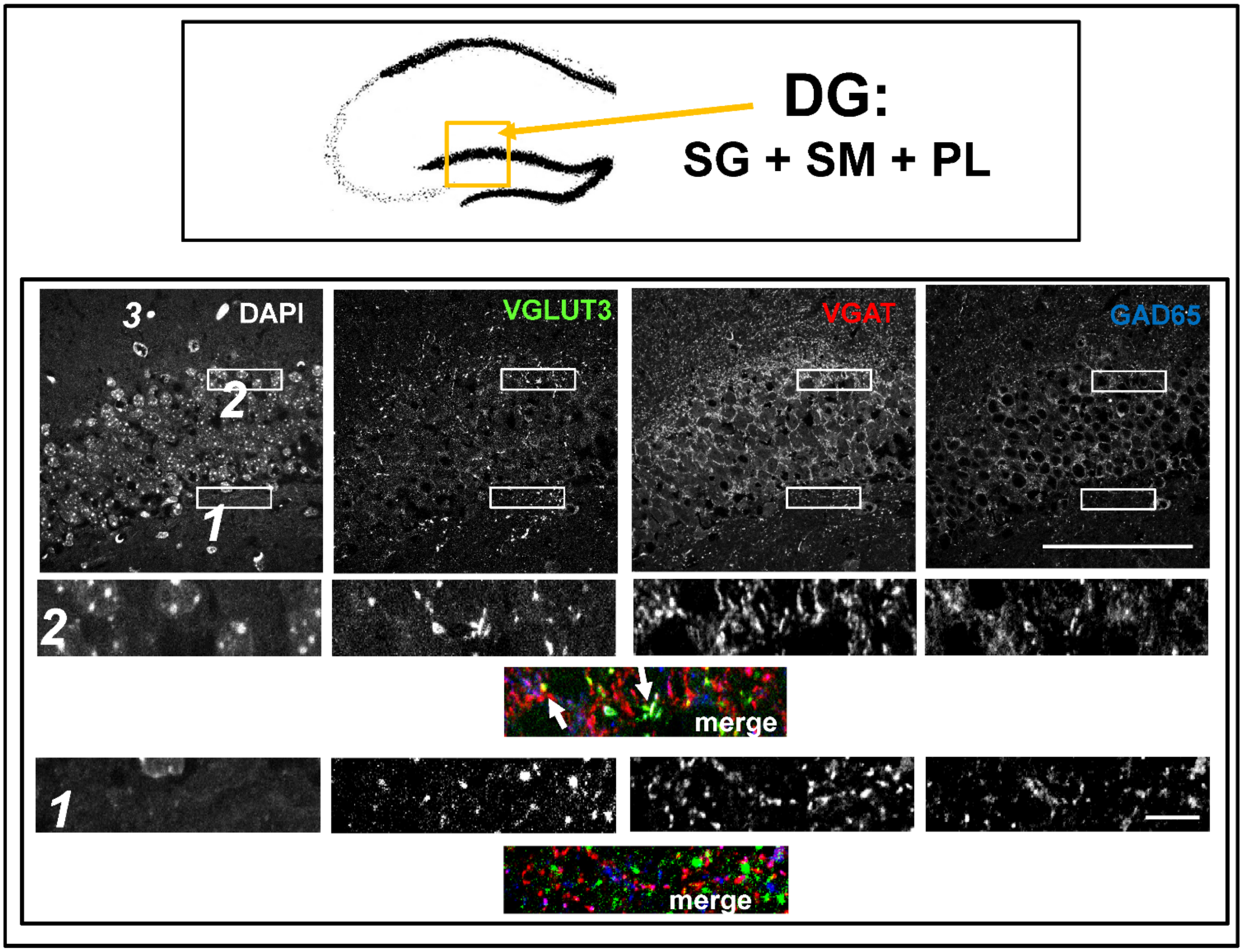

Supplementary Figure S3, 4: Partial colocalization of VGLUT3, Syn1/2, VGAT, and GAD65 puncta in the stratum granulare and in the intragranular mossy fibers of the dentate gyrus.
Top box, Schematic overview of hippocampus with enlarged region (yellow square; here: dentate Gyrus = DG, containing stratum granulare = SG, stratum moleculare = SM, and polymorphic layer = PL). **Bottom box,** confocal immunofluorescence stainings of coronal sections from wild-type mice probed with the VGLUT2 (white; green in merge), VGAT (white; red in merge), and GAD65 (white;

blue in merge) antibodies. The upper row (large squares) shows the enlarged region (3 = SM; 2 = SG; 1 = PL; scale bar = 100 µm), the bottom rows (small rectangles) show further enlargements (indicated in upper row by white rectangles) in the SG (2, top row) and PL (1, bottom row) (scale bar = 5 µm). White arrows indicate selected puncta with visible colocalization of VGLUT3 and VGAT, and partial colocalization with GAD65.

Supplementary Figure S4

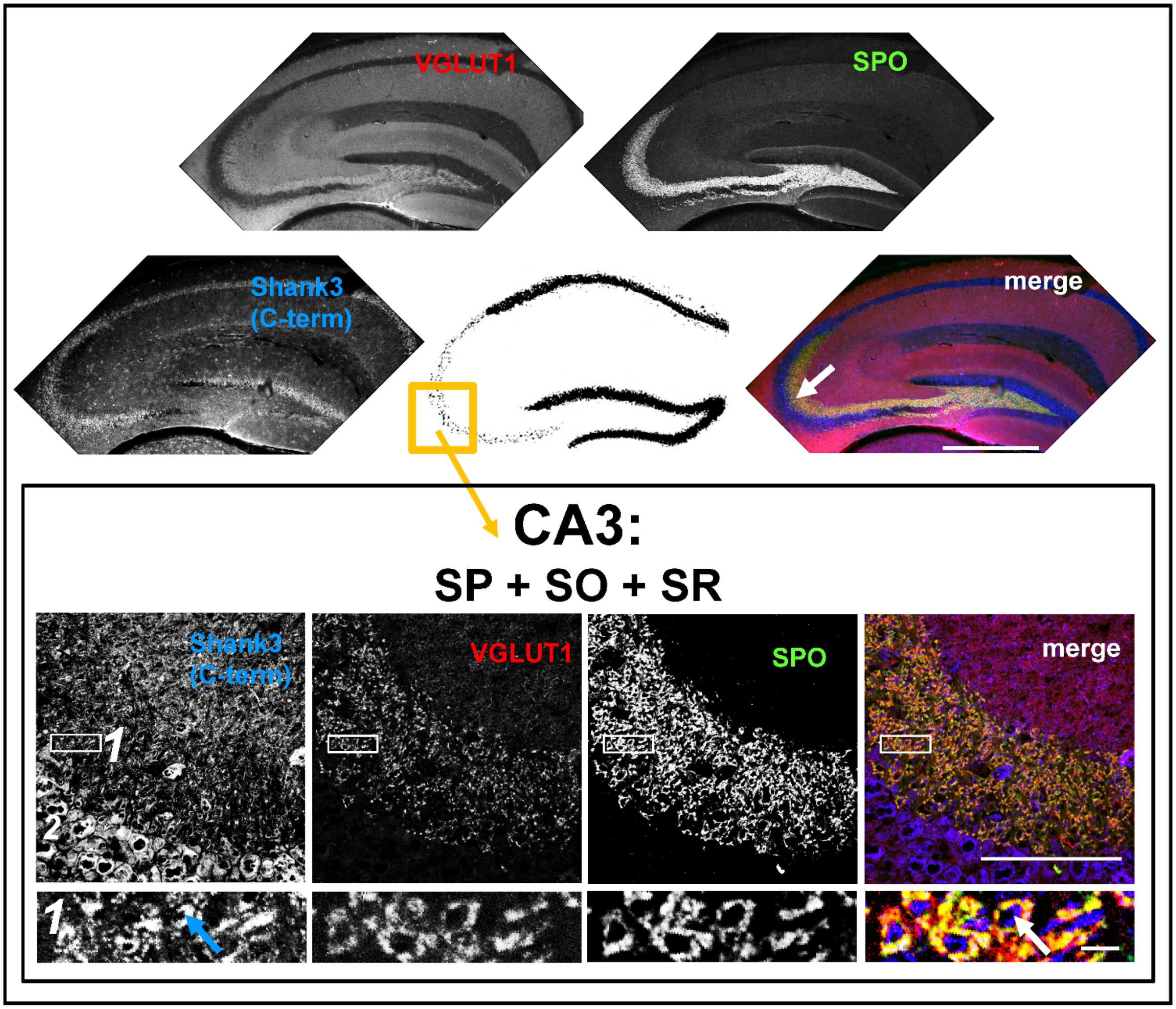

Supplementary Figure S4: Colocalization of Shank3 immunoreactivity with VGLUT1/SPO in the stratum radiatum/stratum lucidum of the CA3 region and partial enclosure of Shank3 by VGLUT1/SPO immunoreactivity.

Top box: Schematic overview of hippocampus with enlarged region (yellow square; here: CA3 containing stratum pyramidale = SP, stratum oriens = SO and stratum radiatum = SR). 5x magnification of hippocampus with immunofluorescence stainings of coronal sections from wild-type mice probed with the „*C-term*“ Shank3 (white; blue in merge), SPO (white; green in merge),

and VGLUT1 (white; red in merge) antibodies. White arrow points towards the CA3 region, which is characterized by a colocalization of SPO and VGLUT1; scale bar (white line) = 300 µm. **Bottom box:** confocal immunofluorescence stainings of coronal sections from wild-type mice probed with the „*C-term*“ Shank3 (white; blue in merge), SPO (white; green in merge), and VGLUT1 (white; red in merge) antibodies. The upper row (large squares) shows the enlarged region (2 = SP; 1 = SR; scale bar = 100 µm), the bottom row (small rectangles) shows a further enlargement (indicated in upper row by white rectangle) in the SR (scale bar = 5 µm). White/blue arrows point towards the subfraction of Shank3 stain, which appears to be enclosed by a colocalized stain of SPO and VGLUT1 which indicates mossy fiber boutons.

Supplementary Figure S5, 1

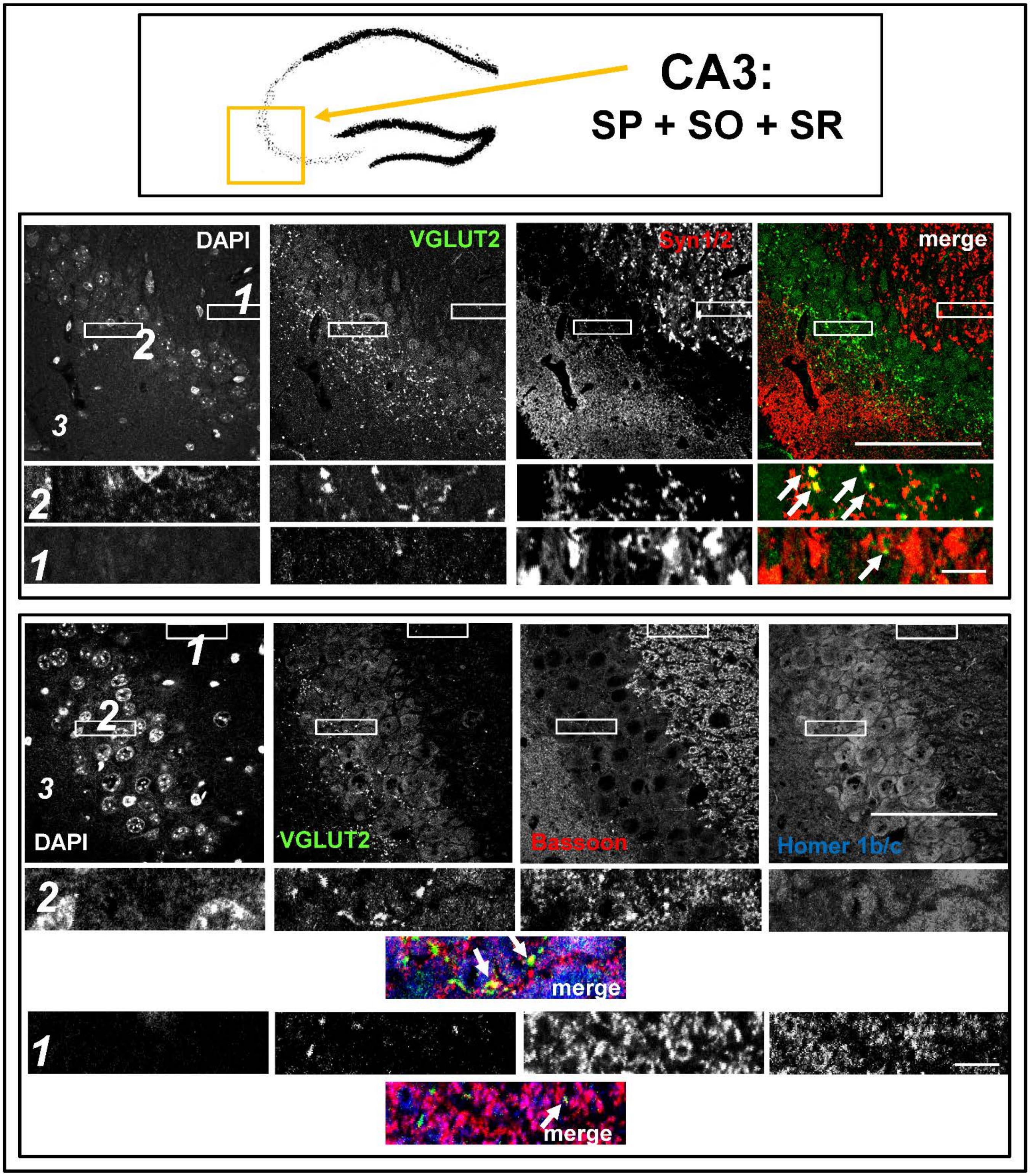

Supplementary Figure S5, 1: Colocalization of VGLUT2 and Syn1/2 puncta in the VGLUT2-band of the stratum pyramidale and in the mossy fiber synapses of the CA3, low colocalization with Bassoon, and no colocalization with Homer 1b/c puncta.

Top box: Schematic overview of hippocampus with enlarged region (yellow square; here: CA3 containing stratum pyramidale = SP, stratum oriens = SO and stratum radiatum = SR). **Middle box:** confocal immunofluorescence stainings of coronal sections from wild-type mice probed with the VGLUT2 (white; green in merge) and Syn1/2 (white; red in merge). The upper row (large squares) shows the enlarged region (3 = SO; 2 = SP; 1 = SR; scale bar = 100 µm), the bottom row (small rectangles) shows further enlargements (indicated in upper row by white rectangles) in the SP (2, top row) and SR (1, bottom row) (scale bar = 5 µm). White arrows indicate selected puncta with visible colocalization of VGLUT2 and Syn1/2. **Bottom box:** confocal immunofluorescence stainings of coronal sections from wild-type mice probed with the VGLUT2 (white; green in merge), Bassoon (white; red in merge), and Homer 1b/c (white; blue in merge) antibodies. The upper row (large squares) shows the enlarged region (3 = SO; 2 = SP; 1 = SR; scale bar = 100 µm), the bottom rows (small rectangles) show further enlargements (indicated in upper row by white rectangles) in the SP (2, top row) and SR (1, bottom row) (scale bar = 5 µm). White arrows indicate selected puncta with visible colocalization of VGLUT2 and Bassoon.

Supplementary Figure S5, 2

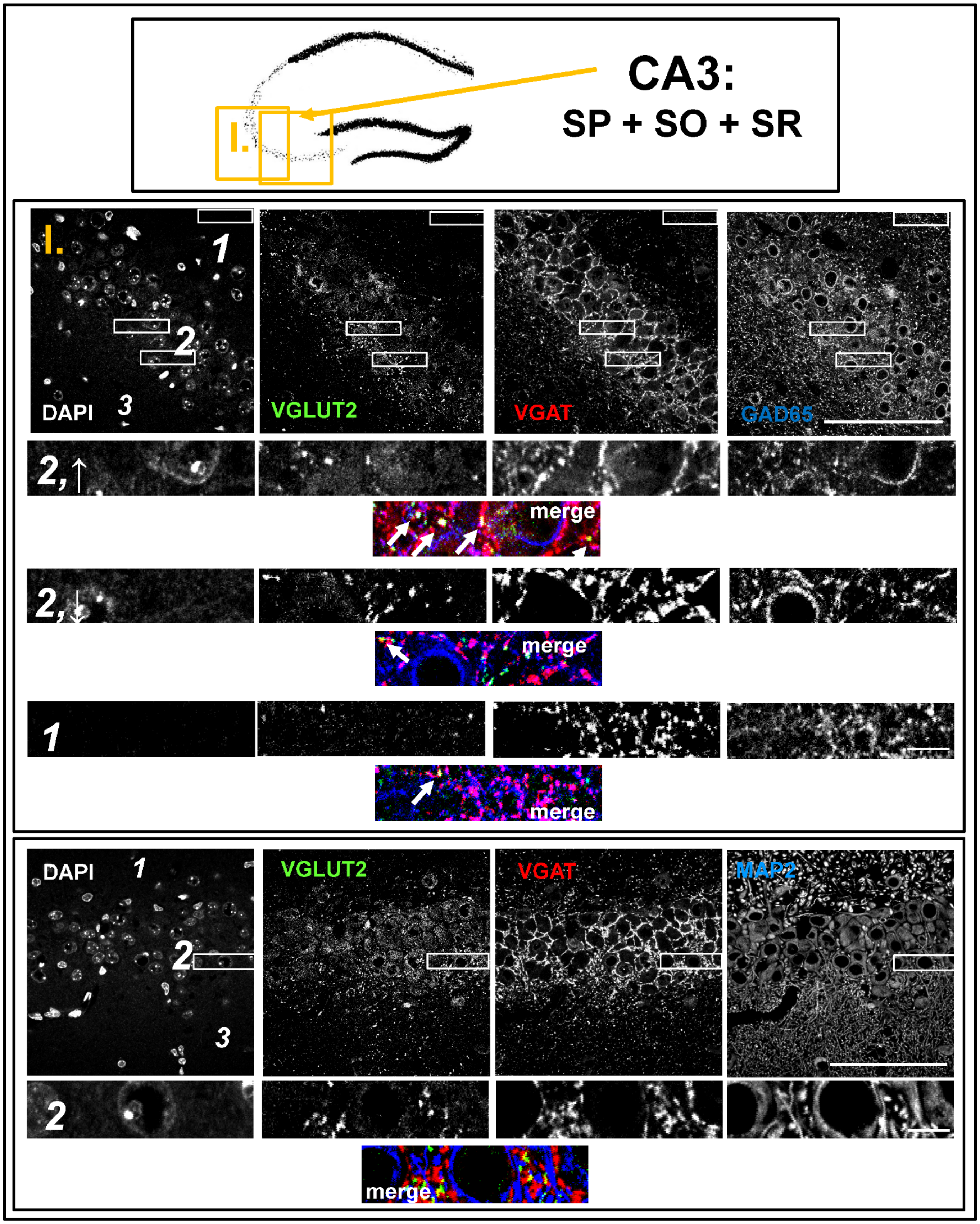

Supplementary Figure S5, 2: Partial colocalization of VGLUT2, VGAT, and GAD65 puncta in the VGLUT2-band of the stratum pyramidale and in the mossy fibers synapses of the CA3.

Top box: Schematic overview of hippocampus with enlarged region (yellow square; here: CA3 containing stratum pyramidale = SP, stratum oriens = SO and stratum radiatum = SR; I. indicates middle box). **Middle box:** confocal immunofluorescence stainings of coronal sections from wild-type mice probed with the VGLUT2 (white; green in merge), VGAT (white; red in merge), and GAD65 (white; blue in merge) antibodies. The uppermost row (large squares) shows the enlarged region (3 = SO; 2 = SP; 1 = SR; scale bar = 100 µm), the bottom rows (small rectangles) show further enlargements (indicated in uppermost row by white rectangles) in the SP (top two rows: 2,↑ indicates upper and 2,↓ indicates lower row) and SR (1, lowest row) (scale bar = 5 µm). White arrows indicate selected puncta with visible colocalization of VGLUT2 and VGAT, and partial colocalization with GAD65. Notice that there are regional differences in the stratum pyramidale in regards to the degree of colocalization of VGLUT2 and VGAT (compare 2,↑ and 2,↓). **Bottom box:** confocal immunofluorescence stainings of coronal sections from wild-type mice probed with VGLUT2 (white; green in merge), VGAT (white; red in merge), and MAP2 (white; blue in merge) antibodies. The upper row (large squares) shows the enlarged region (3 = SO; 2 = SP; 1 = SR; scale bar = 100 µm), the bottom rows (small rectangles) show further enlargements (indicated in upper row by white rectangles) in the VGLUT-2 band in the SR (bottom row) (scale bar = 5 µm).
