# Supplementary figures and images for: Selective Localization of Shanks to VGLUT1-Positive Excitatory Synapses in the Mouse Hippocampus
Source: Front Cell Neurosci. 2016 Apr 26;10:106. doi: 10.3389/fncel.2016.00106 (PMC4844616; doi:10.3389/fncel.2016.00106)

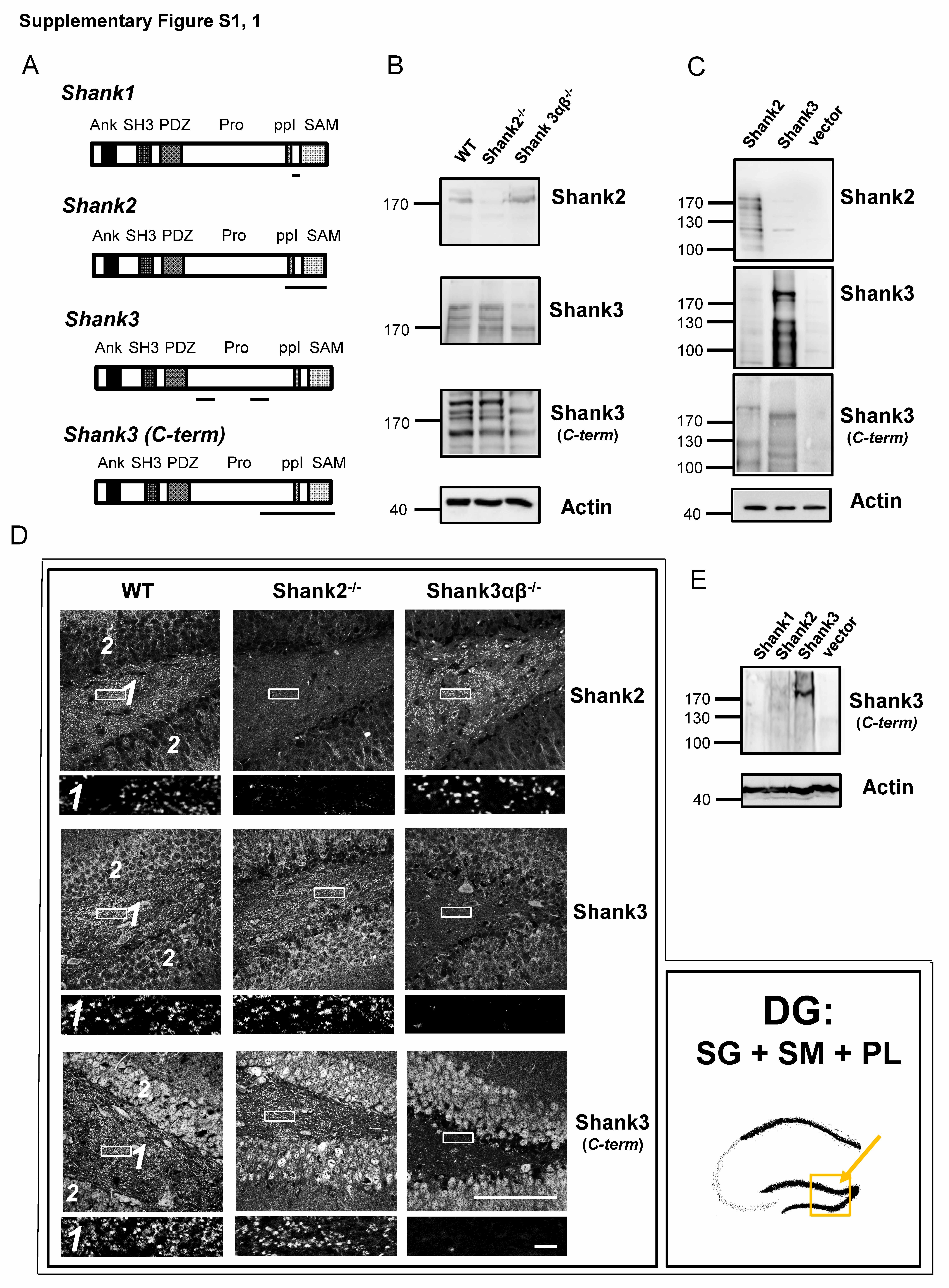

Supplement: Supplementary file 2 [file Image1.JPEG]

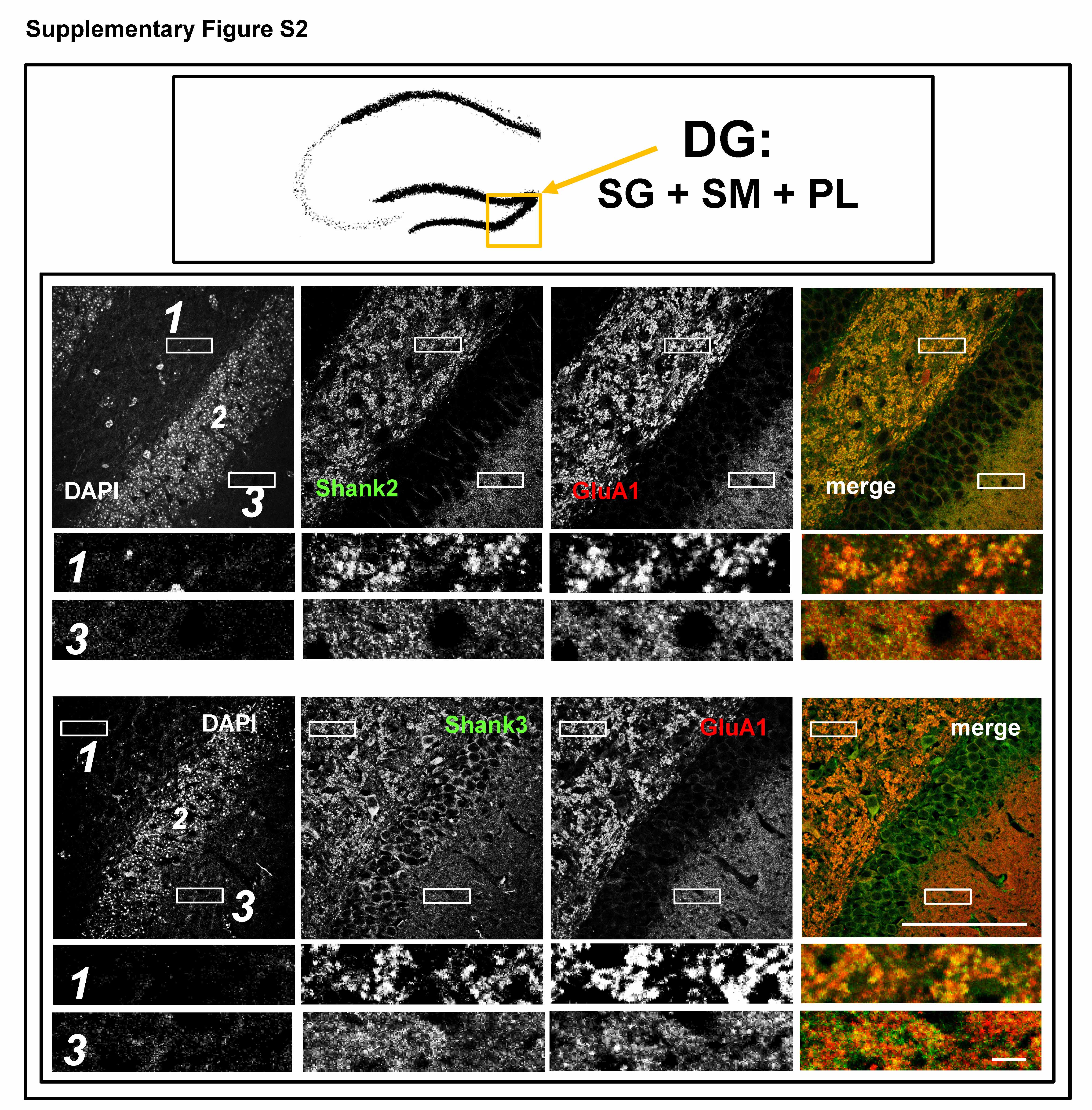

Supplement: Supplementary file 4 [file Image3.JPEG]

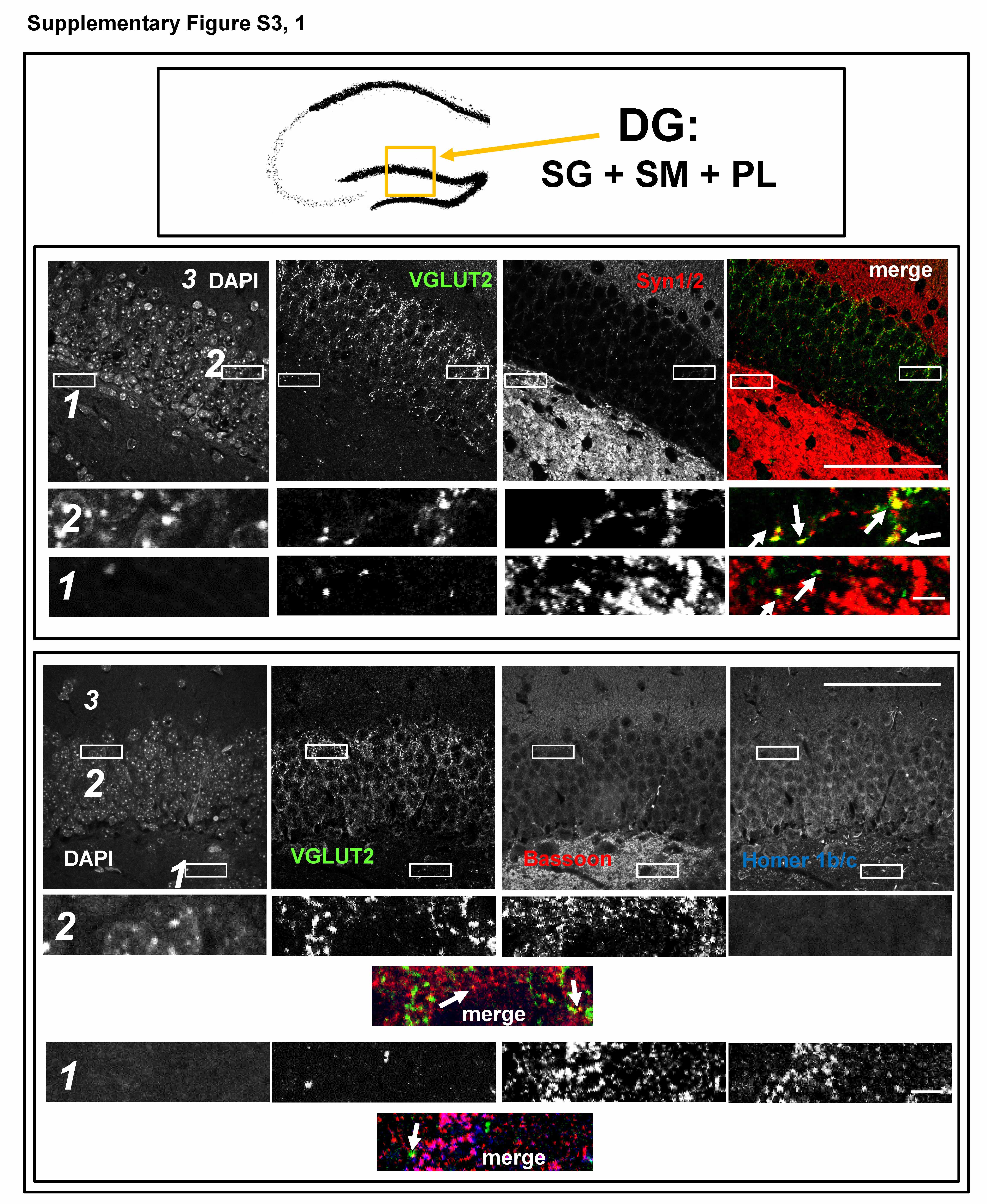

Supplement: Supplementary file 5 [file Image4.JPEG]

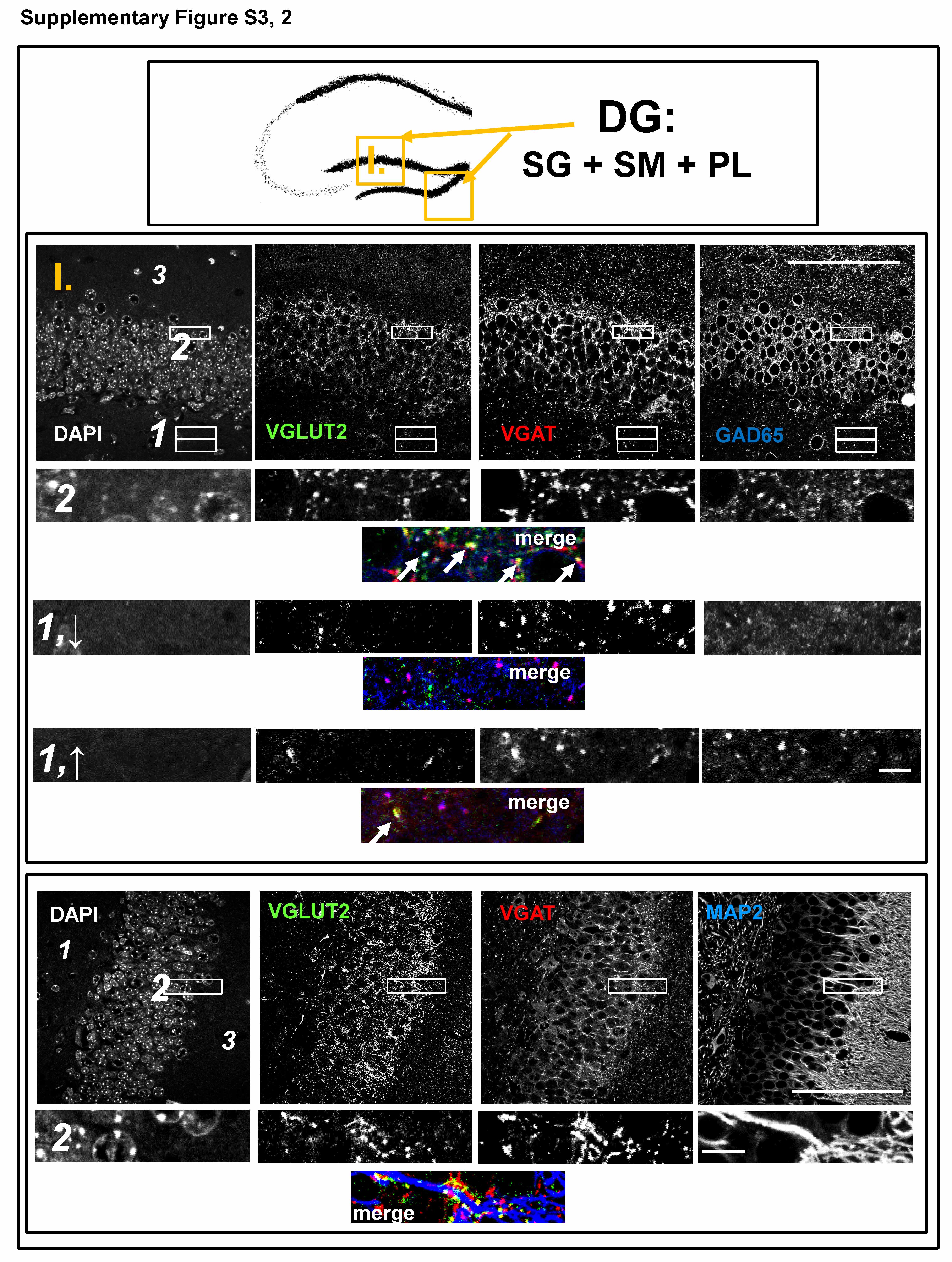

Supplement: Supplementary file 6 [file Image5.JPEG]

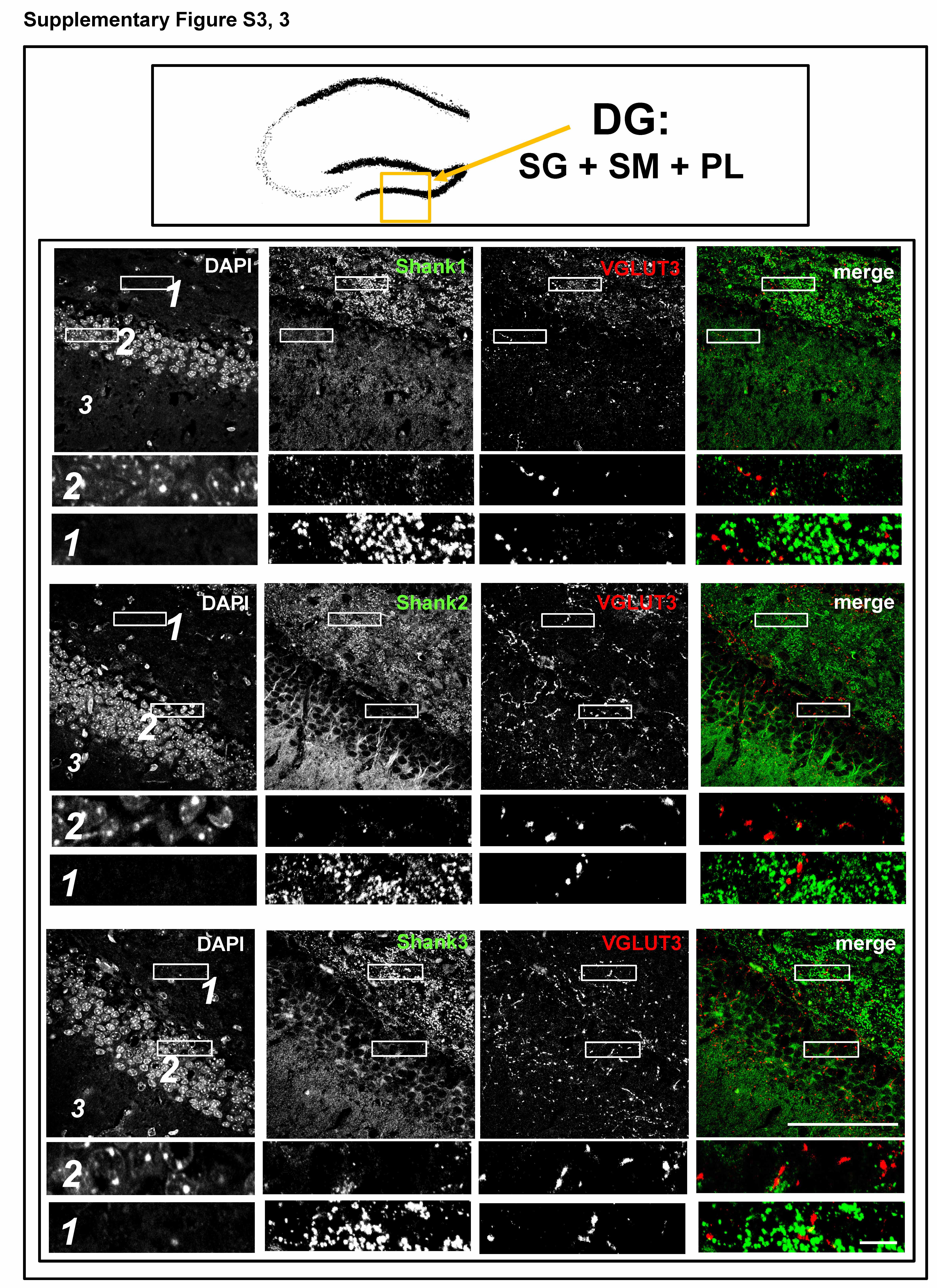

Supplement: Supplementary file 7 [file Image6.JPEG]

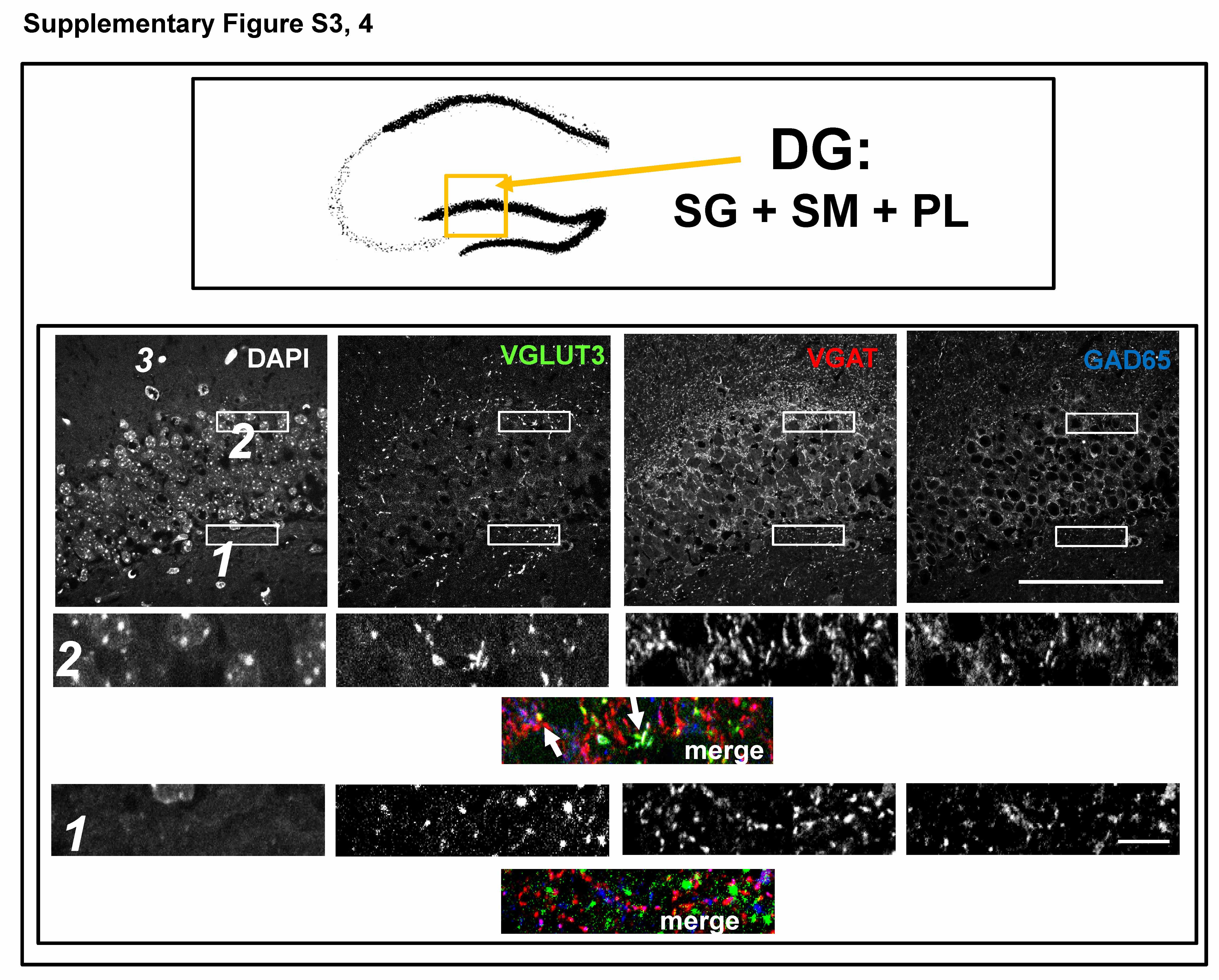

Supplement: Supplementary file 8 [file Image7.JPEG]

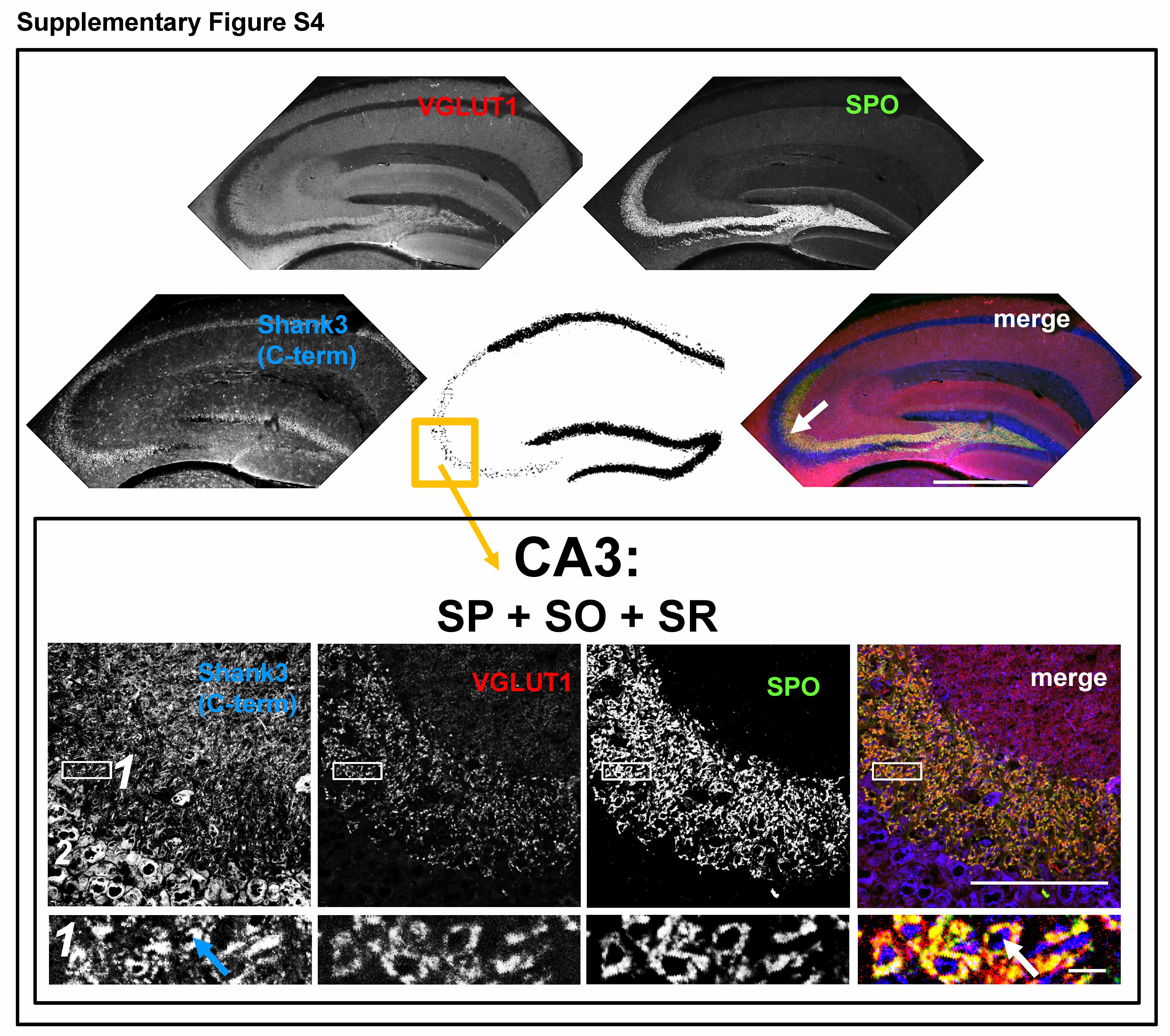

Supplement: Supplementary file 9 [file Image8.JPEG]

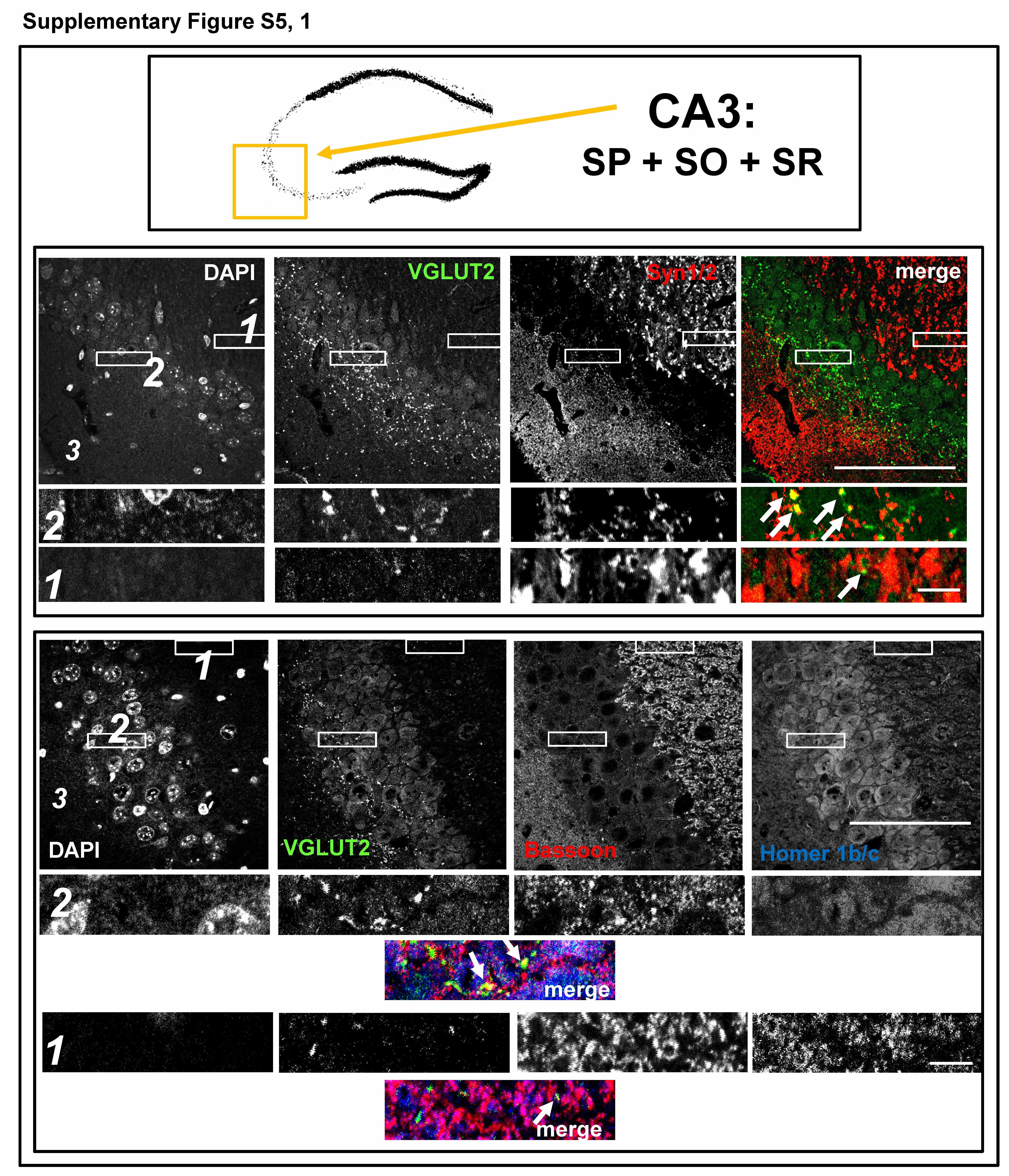

Supplement: Supplementary file 10 [file Image9.JPEG]

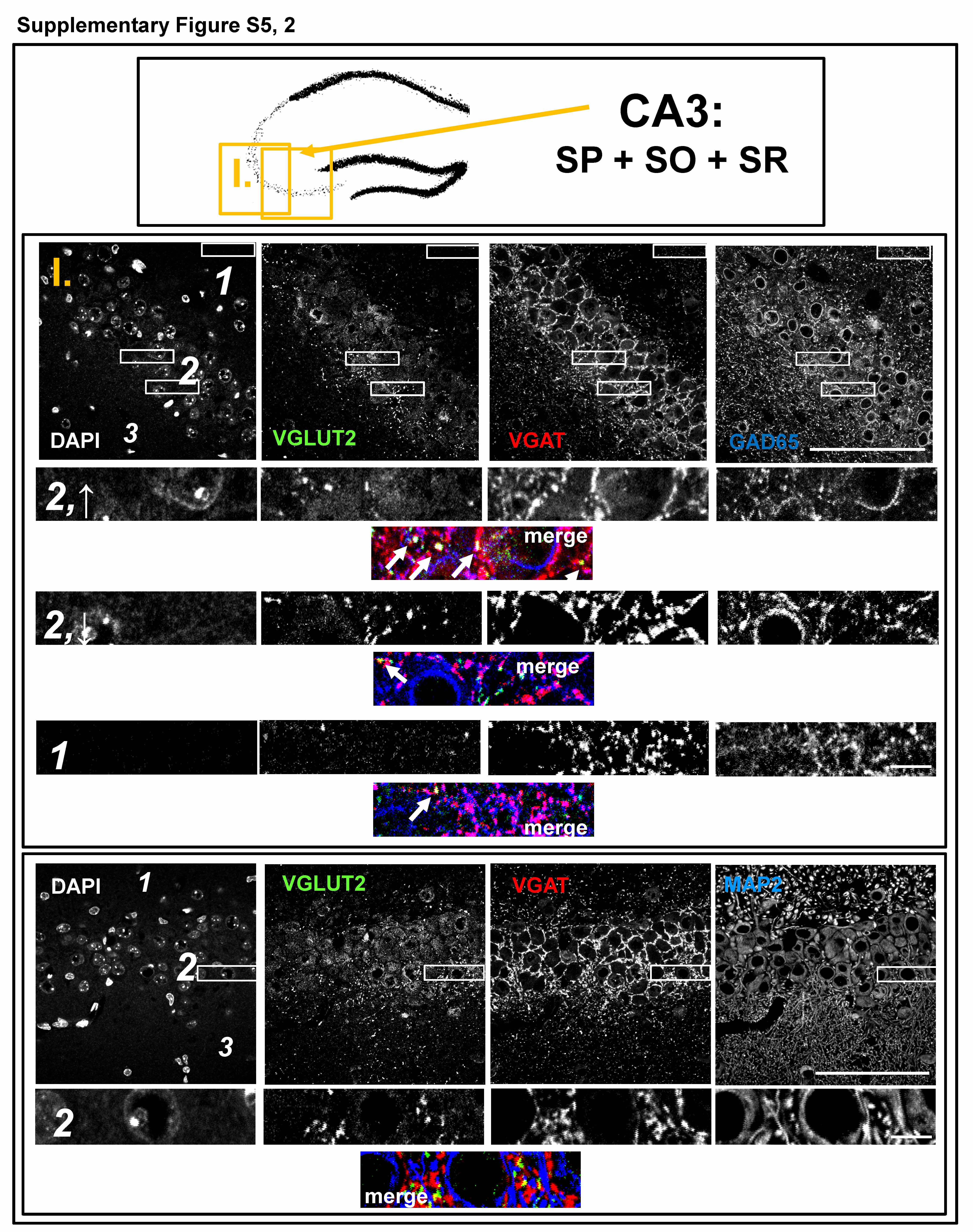

Supplement: Supplementary file 11 [file Image10.JPEG]

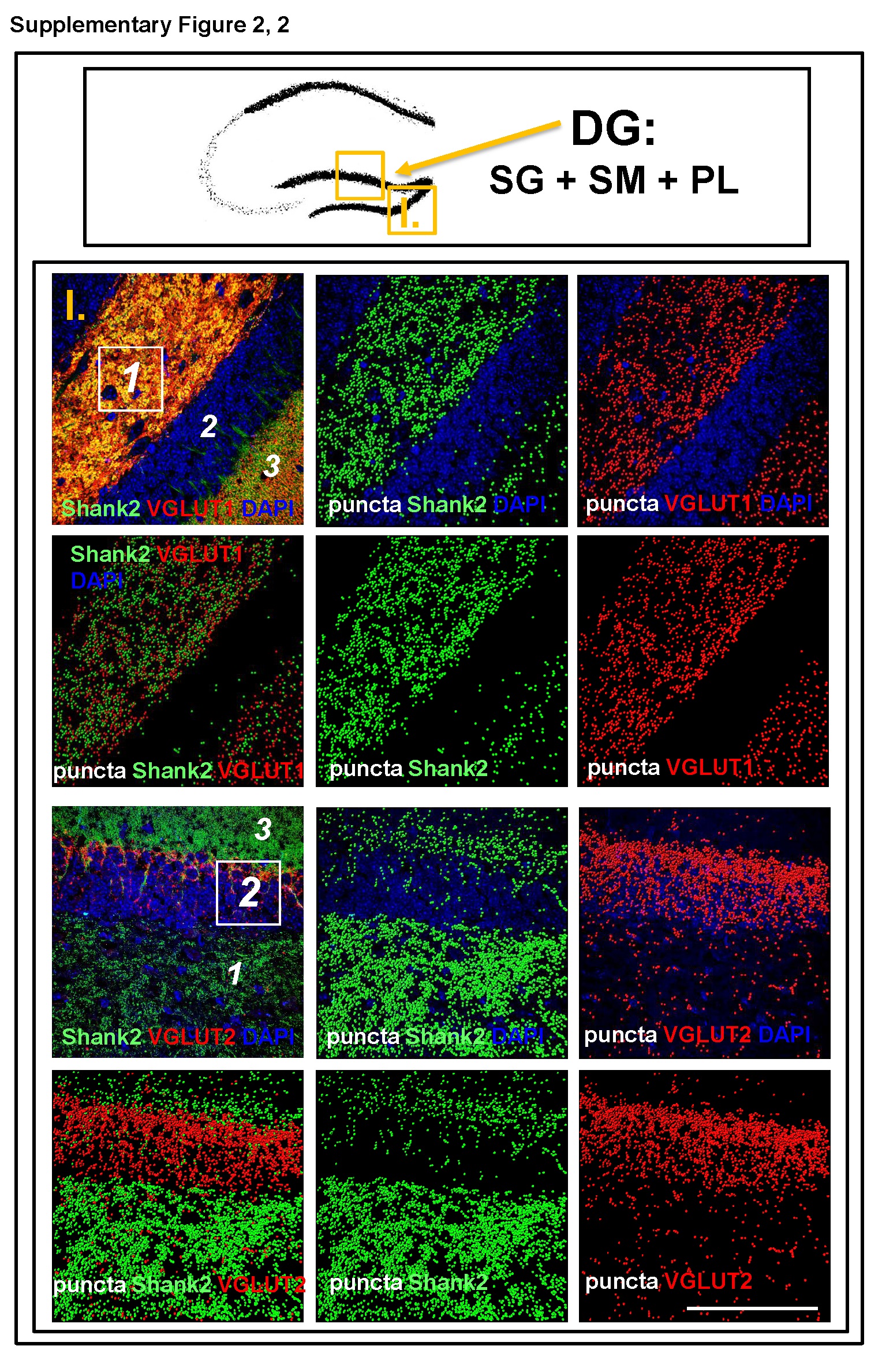

Supplement: Supplementary file 12 [file Image11.JPEG]

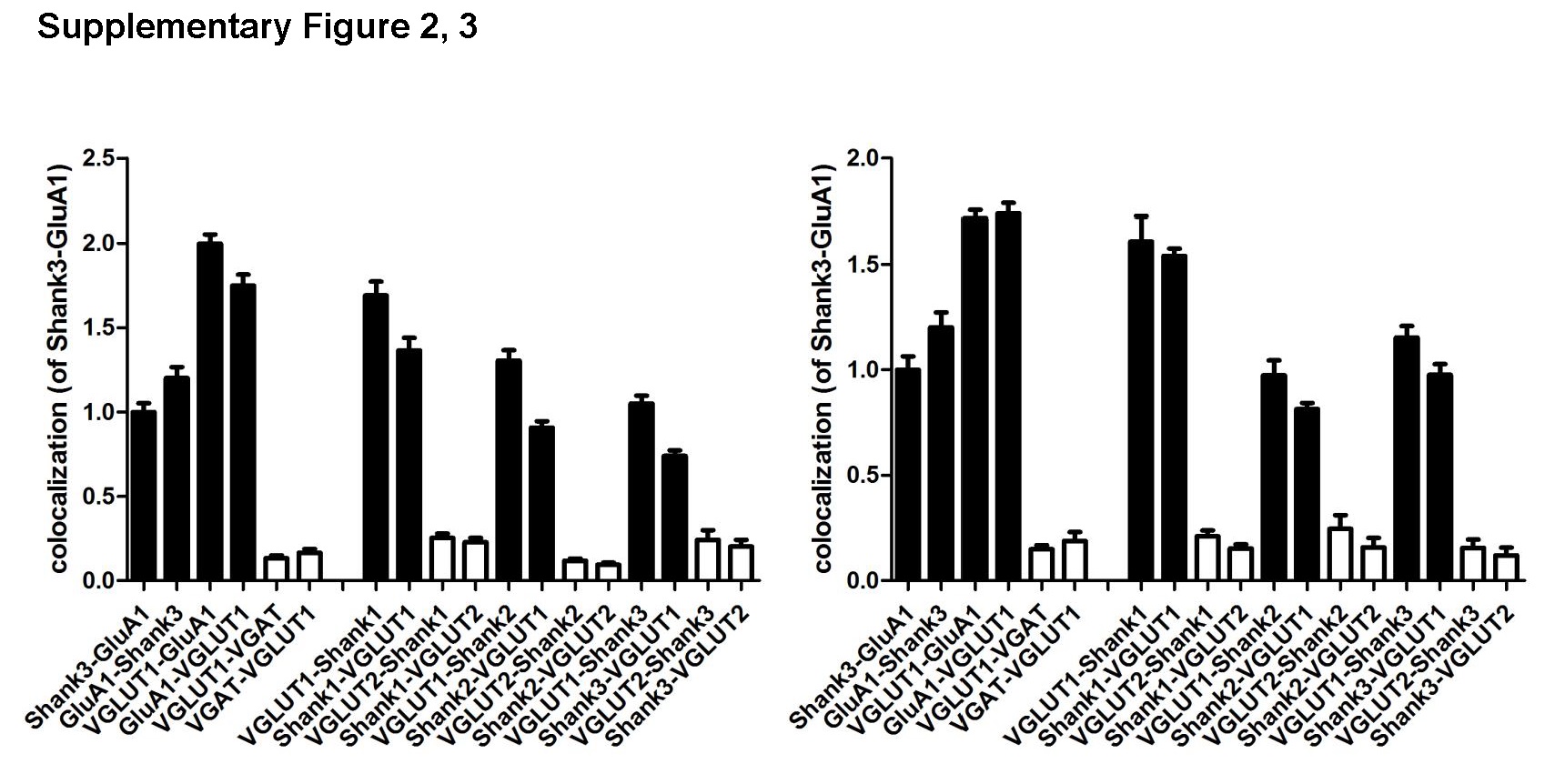

Supplement: Supplementary file 13 [file Image12.JPEG]
